# Structures, Biological Activities, and Food Industry Applications of Anthocyanins Sourced from Three Berry Plants from the Qinghai–Tibet Plateau

**DOI:** 10.3390/foods14213660

**Published:** 2025-10-27

**Authors:** Yaping Luo, Lichengcheng Ren, Shizheng Zhang, Yongjing Xie, Honglun Wang, Na Hu

**Affiliations:** Qinghai Provincial Key Laboratory of Tibetan Medicine Research, CAS Key Laboratory of Tibetan Medicine Research, Northwest Institute of Plateau Biology, Xining 810008, China; ys241055000971@qhu.edu.cn (Y.L.); ys221055001395@qhu.edu.cn (L.R.); zsz415@126.com (S.Z.); xieyongjing24@mails.ucas.ac.cn (Y.X.); hlwang@nwipb.cas.cn (H.W.)

**Keywords:** anthocyanin, berry, Qinghai–Tibet Plateau, chemical structure, biological activity, food applications, *Lycium ruthenicum* Murr., *Nitraria tangutorun* Bobr, *Rubus idaeus*

## Abstract

The distinctive geographical environment of the Qinghai–Tibet Plateau has nurtured a variety of anthocyanin-rich berry plants. This review systematically summarizes the current state of research on anthocyanins obtained from *Lycium ruthenicum* Murr. (LRAs), *Nitraria tangutorun* Bobr (NTAs), and *Rubus idaeus* (RAs) for their potential health benefits and use. The anthocyanins found in these three berries have attracted considerable interest for their significant biological effects, such as their antioxidant, anti-aging, hypoglycemic, anti-inflammatory, and neuroprotective activities, as well as their ability to regulate the gut microbiota and inhibit cancer cells. These anthocyanins have potential applications as natural colorants, packaging materials and smart labels, as well as functional food and health supplements in the food industry. They have diverse molecular architectures with glycosylation and acylation profiles. The structural features of anthocyanins are closely related to their biological activities. This review provides a detailed overview of the chemical structures, synthesis pathways, biological activities, and applications in the food industry of LRAs, NTAs, and RAs. This summary offers a theoretical foundation for exploring plant resources characteristic of the Qinghai–Tibet Plateau and for the development and utilization of high-value-added functional foods, pharmaceuticals, and cosmetics.

## 1. Introduction

The Qinghai–Tibet Plateau, known as the “Roof of the World”, stretches from the Pamir Plateau in the west to the Hengduan Mountains in the east. It is bounded in the north by the Kunlun Mountains, the Altun Mountains, and the Qilian Mountains and in the south by the Himalayas [1]. The Plateau is characterized by rich biodiversity due to its unique geographical and climatic conditions [2]. Among the flora of the region, berry plants are particularly significant due to their broad ecological value and traditional medicinal and nutritional applications. These plants have evolved unique adaptive mechanisms to synthesize vital secondary metabolites under extreme conditions, making them tolerant to high altitudes, hypoxic environments, and intense ultraviolet radiation [3,4].

*Lycium ruthenicum* Murr. (LRM), *Nitraria tangutorun* Bobr (NTB) and *Rubus idaeus* (RI) all belong to the berry family of plants. In China, they are primarily distributed across the northwestern and northeastern regions. They possess ecological characteristics such as drought tolerance, poor fertility, salt–alkali tolerance, and strong soil and water conservation capabilities [5,6,7]. These plants exhibit excellent stress resistance and are particularly suitable for growing in the harsh environment of the Qinghai–Tibet Plateau. As an ecological economic forest plant, their fruits are not only consumed fresh but also commonly processed into juice, jam, fruit wine, health products, cosmetics, and natural pigments [6]. In both traditional medicine and modern research, their fruits are considered to have health benefits [8]. These fruits are rich in trace elements, polyphenols, and flavonoids. They have also garnered attention for their high anthocyanin content and potential for the industrial production of berry anthocyanins [9].

Anthocyanins, a class of water-soluble flavonoids, are widely present in the flowers, fruits, and leaves of plants and exhibit different colors including blue, purple, and red [10]. Black wolfberry (LRM), blueberries, strawberries, raspberries, and cranberries are commonly consumed fruits rich in anthocyanins. Anthocyanidins are the aglycone form, and anthocyanins exist in plants in the form of glycosides formed via the combination of anthocyanidin with one or more sugars by glycosidic bonds [11]. To date, more than twenty types of anthocyanidin core structures have been discovered in nature, with six predominant core structures—petunidin, cyanidin, pelargonidin, malvidin, delphinidin, and peonidin—accounting for approximately 90% of These fruits are rich in trace elements, polyphenols, and flavonoids. They have also garnered attention for their high anthocyanin content and potential for the industrial production of berry anthocyanins all naturally occurring anthocyanins [12,13]. The structures of anthocyanins are relatively diverse. Glycosylation and acylation structures give rise to a variety of derivative products, which not only improve the structural stability of anthocyanins but also increase their bioavailability [14]. Anthocyanins exhibit diverse biological activities, such as antioxidant, anti-aging, hypoglycemic, and cancer cell growth-inhibiting effects [15,16,17,18]. They have become a hot research topic and increasingly attract attention.

At present, there are many reviews focusing on the anthocyanins in blueberries, strawberries, and cranberries. Despite these research efforts, there are few studies summarizing the structures and biological activities of anthocyanins sourced from berry plants from the Qinghai–Tibet Plateau. This review systematically examines the structural characteristics, biogenic synthesis pathways, biological activities, and food industry applications of *Lycium ruthenicum* Murr. anthocyanins (LRAs), *Nitraria tangutorun* Bobr anthocyanins (NTAs), and *Rubus idaeus* anthocyanins (RAs). The aim of this review is to provide scientific data for the further development and utilization of anthocyanins sourced from the berry plants LRM, NTB, and RI.

## 2. Brief Introduction of Three Berry Plants from the Qinghai–Tibet Plateau

From a dietary perspective, LRM, blueberries, strawberries, raspberries, cranberries, and related fruits are recognized as the most abundant sources of anthocyanins [19]. The main anthocyanin types and total anthocyanin contents of these berries are summarized in Table 1. The anthocyanin content of LRM fruit is significantly higher than that of other berry plants, reaching 450–550 mg/100 g FW and earning it the title “the king of anthocyanins” [20]. It is well known that in nature, the majority of anthocyanins are cyanidins [14]. However, the main types of anthocyanins are petunidin compounds, which are very rare in berry plants. Similarly to other berry plants, the anthocyanins in NTB and RI are composed primarily of cyanidins. In terms of substituents, all anthocyanins of the berry plant undergo glycosylation. However, acylation often occurs in the anthocyanins of LRM and NTB, which has not been reported in other plants. It is worth emphasizing that this can not only increase their structural diversity but also enhance the stability of the anthocyanins, which could offer a broader range of applications. Therefore, LRM, NTA, and RA are berries containing anthocyanins with different characteristics.

### 2.1. Lycium ruthenicum Murr.

LRM is a multi-branched thorny shrub belonging to the *Lycium* genus of the Solanaceae family. It is also called “black wolfberry” due to its distinctive black fruit. It is resistant to cold, drought, and saline conditions, among others, and plays an important role in the construction of the ecological environment. LRM is most widely distributed in central Asia, the Caucasus, and Europe. Within China, as a characteristic medicinal plant of the northwestern desert and is mainly distributed in the Xinjiang, Qinghai, Ningxia, Gansu, and Inner Mongolia regions. The Tibetan medicinal name for its fruit is “Punma”, and it is recorded in the classic Tibetan medicinal texts, Four Medical Classic and Jing zhu Ben Cao. Its fruit parts are often used in folk medicine for nourishing, strengthening, and lowering blood pressure [32]. It is worth noting that the anthocyanin content of LRM from the Qinghai region is the highest among all production areas thanks to its adaptation to the extreme environment of the plateau (such as its high altitude and strong ultraviolet rays). The methods of extracting LRA, detailed extraction parameters, and corresponding yields are summarized in Table 2 [33]. The main methods are solvent, ultrasound-assisted, microwave-assisted, and enzymatic extraction, encompassing both traditional and modern approaches. Among these techniques, the highest LRA yield is achieved with ultrasonic-assisted enzymolysis extraction, reaching up to 31.6 mg/g [34].

### 2.2. Nitraria tangutorun Bobr

NTB is a shrub species that is primarily distributed in arid regions, including deserts like the Gobi, and belongs to the genus *Nitraria* within the family Nitrariaceae [5]. Its mature fruit exhibits coloration ranging from light red to dark red and purple. In China, this species is predominantly distributed in northwestern regions, including Gansu, Ningxia, and Qinghai. The Qinghai–Tibet Plateau has a high distribution of NTB [3]. NTB possesses a strong ability to adapt to arid and saline environments, which makes it an ideal plant for windbreaks, sand fixation, and maintaining the ecological balance of desert grasslands [5]. In addition to its significant ecological value, the fruit has long been valued for its medicinal properties. The Compendium of Materia Medica describes NTB fruit as possessing a sweet–sour taste, non-toxic characteristics, and the ability to ameliorate spleen–stomach deficiency, poor appetite, and diarrhea, as well as strengthen the body, enhance physical health, and brighten the eyes through long-term intake. Methods of extracting anthocyanins from NTB fruit include aqueous two-phase, ultrasound-assisted and ultrasound-assisted deep eutectic solvent extractions (Table 2). Among these approaches, microwave/ultrasound-assisted enzymatic extraction demonstrates the highest extraction efficiency, extracting 3.862 mg/g NTAs from dried fruit [44].

### 2.3. Rubus idaeus

RI, commonly referred to as raspberry, is a deciduous shrub belonging to the genus *Rubus* within the family Rosaceae. The species is widely distributed across temperate and boreal regions globally, and in China, its distribution is primarily concentrated in the northeastern, northwestern, and northern regions. In traditional medicine, RI has been recognized for its therapeutic potential, and it has historically been employed in the prevention of various conditions, including infertility, impotence, low backache, poor eyesight, and frequent urination [45]. With ongoing optimization of extraction processes, the yield of RA has been progressively improved using ultrasound-assisted or microwave-assisted extraction (Table 2). Microwave-assisted extraction achieves the highest yield of RA, reaching 2.18 mg/g [43].

The above section systematically introduces the botanical characteristics, geographical distribution, traditional uses, and methods of extracting anthocyanins from three anthocyanin-rich berry plants. As a key secondary metabolite that helps plants resist adverse conditions, such as strong ultraviolet radiation and drought, there is dual ecological and economic value in developing and utilizing anthocyanins from three berry plants have dual ecological and economic value.

## 3. Chemical Structures of Anthocyanins from Three Berry Plants

**Figure 1:**
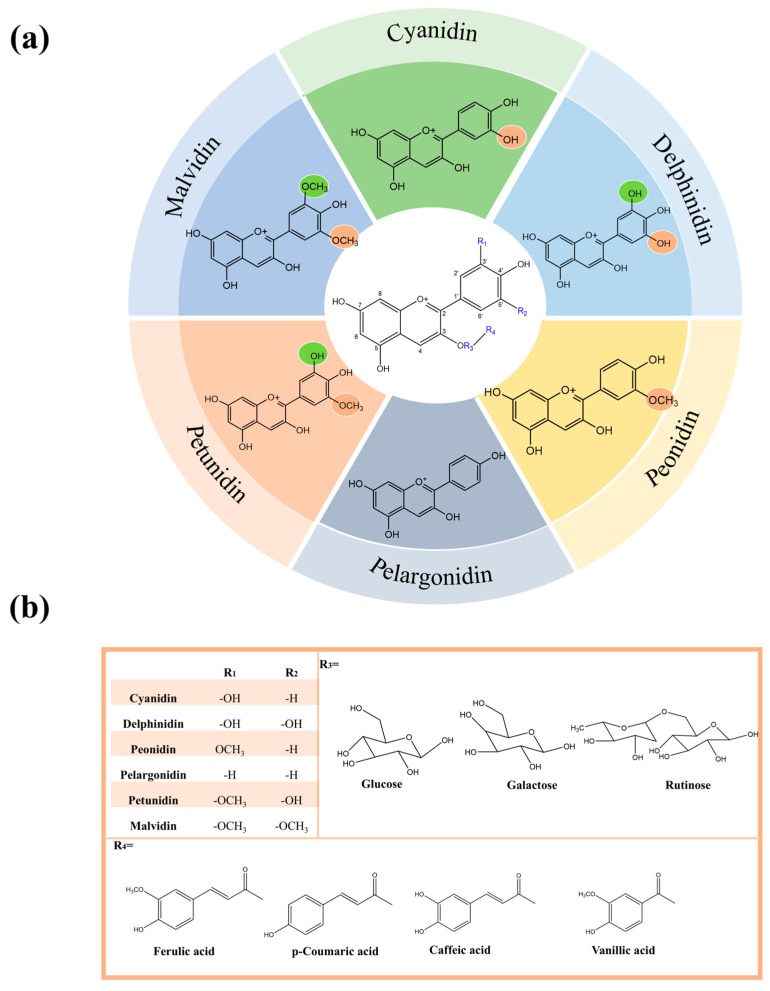
(**a**) Anthocyanin core structure; (**b**) the primary structural substitutions of anthocyanins. (This figure was originally created by the author).

Anthocyanins are a class of polyhydroxy compounds with a flavonoid structure. The fundamental core structure is characterized as a 2-phenylbenzopyran cation consisting of two benzene rings connected by an oxygenated heterocycle, with a backbone structure of C6-C3-C6. Specifically, when no substitution occurs at positions 3′ and 5′, it is a pelargonidin anthocyanidin. When the 3′ position is replaced by hydroxyl or methoxy groups, cyanidin or peonidin anthocyanidin, respectively, is formed. Delphinidin forms when the hydroxyl substituent occurs in both the 3′ and 5′ positions, while malvidin forms when the methoxy substituent occurs in both the 3′ and 5′ positions. In addition, when the 3′ position is substituted by methoxy and the 5′ position is substituted by hydroxyl, petunidin is formed (Figure 1).

### 3.1. Anthocyanins Structures of LRM

**Figure 2:**
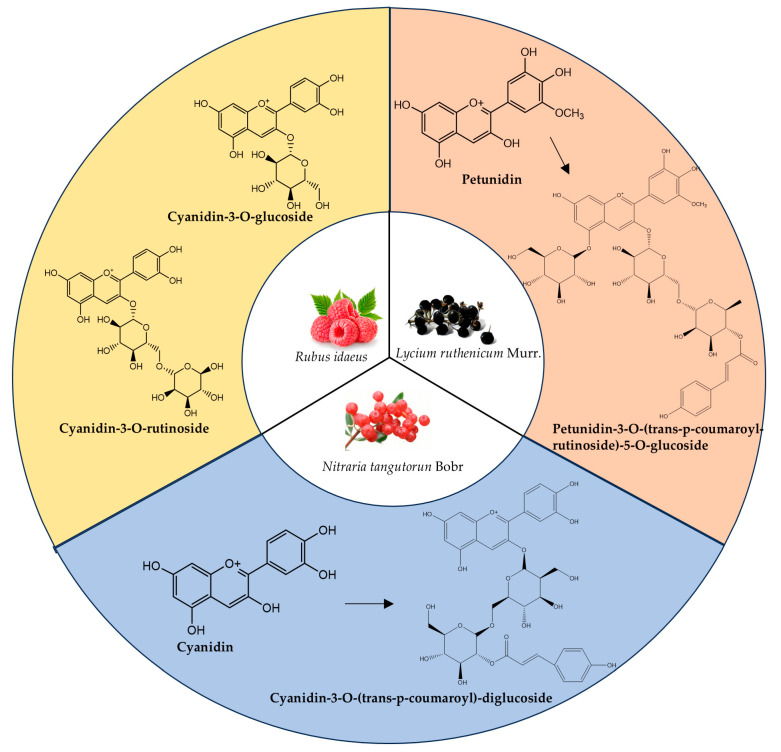
The main anthocyanin structure types in LRM, *Nitraria tangutorun* Bobr (NTB), and *Rubus idaeus* (RI) fruits. (This figure was originally created by the author).

LRMs contain abundant anthocyanins, which are the primary basis for their multiple pharmacological activities. LRAs comprise multiple types of anthocyanins, including petunidin, delphinidin, pelargonidin, and malvidin derivatives, with petunidin derivatives constituting over 95% of the total content [46]. Petunidin derivatives account for the majority of LRAs. Glycosylation is a key structural feature of anthocyanins, and glycosyl substitutions usually occur at the 3 and 5 positions in LRAs. The sugars substituted at the 3-position are mostly glucose, rutinose, and galactose, whereas the sugar substitution at the 5-position is mainly glucose. The glycosyl moieties of LRAs are often further acylated with aromatic acids such as coumaric acid, caffeic acid, and ferulic acid. An acylation group can improve stability by increasing the water solubility of an LRA and forming an intramolecular H-bonding network within the anthocyanin molecule [14,47]. The types of LRAs reported in this article are summarized in Table 3. Notably, petunidin-3-O-(trans-p-coumaroyl-rutinoside)-5-O-glucoside accounts for approximately 80% of LRAs [21] (Figure 2). This predominance of a single anthocyanin species is uncommon in nature and may confer UV-B resistance advantages. Furthermore, this chemical specificity facilitates compound isolation and presents significant industrial potential for anthocyanin purification.

### 3.2. Anthocyanin Structure of NTB

Although NTAs include cyanidin-, malvidin-, peonidin-, pelargonidin-, and delphinidin-based derivatives, unlike LRAs, cyanidin-based derivatives are the main anthocyanins in NTAs, the content of which was found to be over 80% of the total anthocyanins in our previous study [23]. Glycosyl substitutions only occur at position 3 in NTAs, and the sugar types are mainly glucose, diglucose, hexose, sambubioside, and pyranose. The acids involved in the acylation of NTAs are mostly coumaric acid, caffeic acid, and ferulic acid. Information about NTAs is provided in Table 4. Among them, cyanidin-3-O-(trans-p-coumaroyl)-diglucoside is the most abundant anthocyanin of NTAs [23] (Figure 2).

### 3.3. Anthocyanin Structure of RI

Raspberries have received extensive research attention due to their delicious taste and vivid pigmentation. RAs are mainly cyanidin and pelargonidin derivatives. These stable molecular configurations are formed through the conjugation of the anthocyanidin aglycone core with various glycosyl moieties, including glucosyl, rutinose, and sophorose residues. However, RA acylation has not been reported yet. Information about RAs is summarized in Table 5. The constituents isolated from RAs have been characterized as predominantly cyanidin-type compounds [55], with cyanidin-3-O-glucoside and cyanidin-3-O-rutinoside identified as the major components (Figure 2), along with trace quantities of pelargonidin and delphinidin derivatives [42]. RI possesses a variety of colors, with fruit color determined by the pH at which the anthocyanins are present [56]. The berry appears red under acidic conditions, and its color gradually changes to purple or blue as the pH increases [57]. These color change characteristics make it suitable as a natural pigment in food, beverage, and other fields, where its application can not only add color to the product but may also contribute some nutritional and health benefits.

In summary, there are significant differences in anthocyanin structure among these three berries, apart from variations in aglycones. LRAs undergo glycosylation at both positions 3 and 5, and the glycosylation at position 3 is often acylated by aromatic acids such as coumaric acid. NTA glycosylation usually only occurs at position 3, but there are various types of sugar chains, and acylation modification also occurs. RAs possess a relatively simple structure that is representative of the anthocyanins in most berry plants.

## 4. Anthocyanin Biosynthetic Pathway

**Figure 3:**
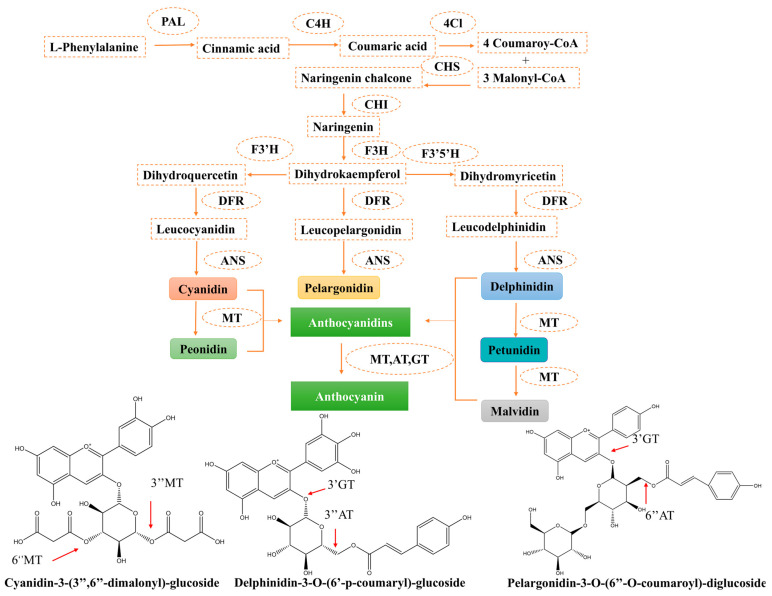
Biosynthesis of anthocyanins. (This figure was originally created by the author.) Notes: PAL, phenylalanine ammonia-lyase; C4H, cinnamic acid 4-hydroxylase; 4CL, 4-coumarate coenzyme A ligase; CHS, chalcone synthase; CHI, chalcone isomerase; F3H, flavanone 3-hydroxylase; F3′H, flavonoid 3′-hydroxylas; F3′5′H, flavonoid 3′,5′-hydroxylase; DFR, dihydroflavonol 4-reductase; ANS, anthocyanidin synthase; MT, methyltransferases; GT, glycosyltransferases; AT, acyltransferases.

The main synthesis pathway for the six most common anthocyanins is as follows [60]: Phenylalanine is the initial product of anthocyanin synthesis [61]. The synthetic pathway comprises three principal stages (Figure 3). First, phenylalanine is converted to cinnamic acid by phenylalanine ammonia-lyase, and the cinnamic acid is subsequently converted to coumaric acid by cinnamic acid 4-hydroxylase. Then, 4-coumaroyl-coenzyme A is formed by the catalyzation of 4-coumarate coenzyme A ligase. In the second stage, 4-coumaroyl-CoA and three molecules of malonyl-CoA are catalyzed by chalcone synthase to form naringenin chalcone, which is further converted to naringenin by chalcone isomerase. The naringenin undergoes hydroxylation at different positions via flavanone 3-hydroxylase, flavonoid 3′-hydroxylase, and flavonoid 3′,5′-hydroxylase to generate dihydrokaempferol, dihydroquercetin, and dihydromyricetin, respectively. Subsequently, three dihydroflavonols are catalyzed by dihydroflavonol 4-reductase to produce colorless precursors, which are then catalyzed by anthocyanidin synthase to produce colored anthocyanidins such as cyanidin, pelargonidin, and delphinidin. Furthermore, cyanidin can be converted into peonidin, and delphinidin can be transformed into petunidin and malvidin with the assistance of methyltransferases [62]. Finally, these colored anthocyanidins react with monosaccharides or polysaccharides and are converted to anthocyanins by glycosyltransferases [63]. The glycosylated anthocyanins are acylated with one or more organic acid molecules via ester bonds. This step is usually mediated by acyltransferases [64]. The glycosylation and acyl structure make these anthocyanins more stable. Anthocyanin biosynthesis is regulated through complex mechanisms influenced by multiple factors. These include environmental stimuli (such as light intensity, temperature fluctuations, and drought stress) and transcriptional regulators (particularly the MYB, bHLH, and WD40 protein families) [65]. Numerous studies have shown that anthocyanins accumulate when plants are subjected to environmental stresses. The synergistic regulation of AtCHS (chalcone synthase) expression mediated by combined UV-A, UV-B, and blue light irradiation has been demonstrated to enhance anthocyanin accumulation [66]. In addition, overexpression of UDP-glycosyltransferases enhanced plant tolerance to low temperature, drought, and salt stress by regulating anthocyanin accumulation [67]. Under extreme stress conditions, reactive oxygen species (ROS) are produced by plants and function as signaling molecules to activate stress tolerance mechanisms. ROS overproduction causes oxidative damage to plants. Research has demonstrated that over production of anthocyanin pigment 1 induces anthocyanin biosynthesis, thereby enhancing the plant’s capacity to mitigate ROS accumulation [68]. The complete biosynthetic pathway and intricate regulatory network of anthocyanins, ranging from primary metabolites to complex stable compounds, have been summarized. GT and AT play a crucial role in the stability and diversity of anthocyanins. Anthocyanins are not simply metabolic byproducts, but key protective agents for plants to actively respond to environmental challenges. This provides a theoretical basis for understanding the differences in anthocyanin content in plants under different growth conditions.

## 5. Biological Activities of Anthocyanins from Three Berry Plants

**Figure 4:**
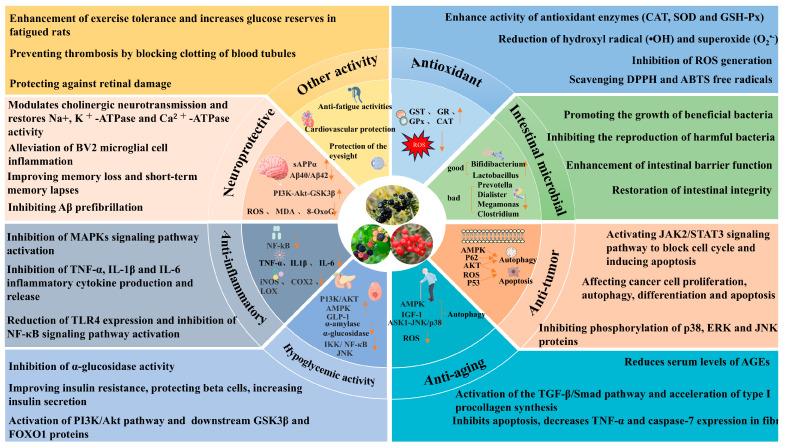
Biological activities of anthocyanins from three berry plants: LRM, NTB, and RI. (This figure was originally created by the author.) Notes: ATPase, adenosine Triphosphatase; Aβ, amyloid β-protein; MAPK, mitogen-activated protein kinase; TNF-α, tumor necrosis factor-α; Il-1β, interleukin-1β; IL-6,interleukin-6; TLR4, toll-like receptor 4; NF-κB, nuclear factor-κB; PI3K/Akt, phosphatidylinositol 3-kinase/protein kinase b; GSK3β, glycogen synthase kinase 3β; FOXO1, forkhead box O1; SOD, superoxide dismutase; CAT, catalase; GSH-Px, glutathione peroxidase; ·OH, hydroxyl radical; O_2_^•−^, superoxide radical; DPPH, 1,1-diphenyl-2-picrylhydrazyl; ABTS, 2,2’-azino-bis (3-ethylbenzothiazoline-6-sulfonic acid); ROS, reactive oxygen species; JAK2/STAT3, janus kinase 2/signal transducer and activator of transcription 3; p38, p38 mitogen-activated protein kinase; ERK, extracellular signal-regulated kinase; JNK, c-jun n-terminal kinase; AGEs, advanced glycation end products; TGF-β, transforming growth factor-β; GR, Glutathione Reductase; p53,tumor protein p53; p62, sequestosome 1; IGF-1, insulin-like growth factor 1; ASK1, apoptosis signal-regulating kinase 1; GLP-1, glucagon-like peptide-1; IKK, iκb kinase; iNOS, inducible nitric oxide synthase; COX-2, cyclooxygenase-2; LOX, lipoxygenase; MDA, malondialdehyde; 8-OxoG, 8-oxoguanine; Aβ40, amyloid-β 40; Aβ42, amyloid-β 42; sAPPα, soluble amyloid precursor protein α.

Anthocyanins have attracted extensive attention due to their remarkable health-promoting properties. An increasing number of studies have shown that anthocyanins exhibit multiple pharmacological activities. Therefore, this review systematically summarizes the pharmacological properties of anthocyanins derived from three berry plants grown on the Qinghai–Tibet Plateau (Table 6), focusing on their antioxidant, hypoglycemic, anti-tumor, anti-aging, anti-inflammatory, neuroprotective, and gut microbiota-regulating effects (Figure 4).

### 5.1. Antioxidant Activity

Oxidative stress is a pathophysiological state characterized by an imbalance of natural antioxidants and free radicals in the body. This condition has been linked to the pathogenesis of multiple chronic health conditions such as atherosclerosis, cancer, neurodegenerative disorders, and coronary artery disease [102,103]. Therefore, the timely supplementation of exogenous antioxidants is an important factor in preventing diseases and maintaining health. Anthocyanins can effectively eliminate free radicals, reduce reactive oxygen species (ROS) levels, and increase levels of antioxidant enzymes such as superoxide dismutase (SOD), catalase (CAT), and glutathione peroxidase (GSH-Px) [102]. In addition, anthocyanins can upregulate the nuclear factor erythroid 2-related factor 2/heme oxygenase-1 (Nrf2-HO-1) signaling pathway to alleviate oxidative stress [69]. Numerous studies have reported that LRAs can effectively reduce levels of 1,1-diphenyl-2-picrylhydrazyl (DPPH), 2,2′-azino-bis (3-ethylbenzothiazoline-6-sulfonic acid) (ABTS), hydroxyl radical (·OH), and superoxide radical (O_2_^•−^), exerting excellent antioxidant effects [21,36]. Moreover, LRAs dose-dependently enhanced the activities of antioxidant enzymes (CAT, SOD, and GSH-Px), attenuated lipid peroxidation injury to PC12 cells, and inhibited H_2_O_2_-induced reactive oxygen cells [70]. Similarly, NTAs and RAs were found to exhibit strong DPPH, ABTS, ·OH, and O_2_^•−^ radical scavenging capacity [41,53]. NTAs could increase SOD activity and total antioxidant capacity (TAC) in hyperlipidemic rats, thereby improving lipid metabolism deficits associated with oxidative stress in a rat model [71]. In addition, RAs were found to inhibit acrylamide-induced oxidative stress in Caco-2 cells by scavenging intracellular ROS, attenuating mitochondrial membrane collapse, and preventing GSH depletion [104]. Moreover, RAs exhibited a stronger ability to protect against cell damage after gastrointestinal digestion [104]. Similarly, long-term administration of LRAs significantly improved hepatic antioxidant capacity in mice [72]. The above studies show that, as natural plant pigments, anthocyanins have enormous antioxidant potential, which may indicate promising prospects for human health development. Oxidative stress is closely related to the occurrence and development of chronic diseases, and antioxidant activity is the most significant biological activity of anthocyanins. Thus, anthocyanins are promising natural agents for preventing and managing various chronic diseases. However, more clinical trials and data are needed to evaluate their antioxidant benefits for the human body.

### 5.2. Anti-Tumor Activity

**Figure 5:**
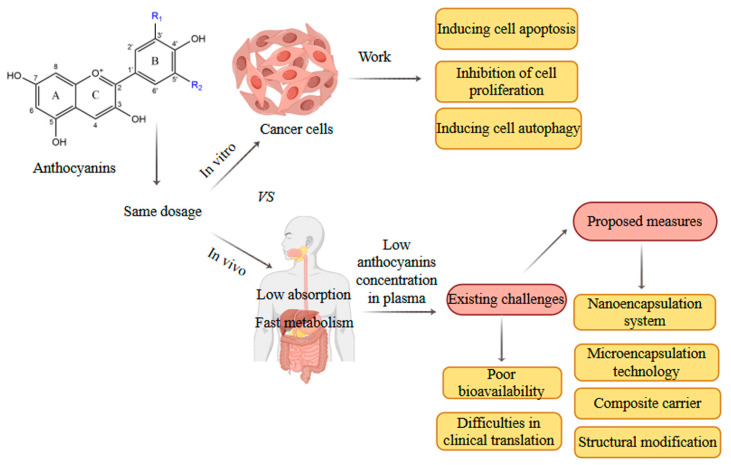
Issues in vitro and in vivo anti-cancer research on anthocyanins and possible solutions. (This figure was originally created by the author).

Tumors remain one of the most challenging global health burdens worldwide. Current standard therapeutic approaches primarily involve radiotherapy and chemotherapy. However, most conventional chemotherapeutic drugs are associated with substantial adverse effects and limited therapeutic efficacy [105,106,107]. Therefore, the search for natural anti-tumor, low-toxicity medicines has become a research hot spot. Anthocyanins, a class of natural plant pigments, have been proven to exhibit significant efficacy in the prevention of tumors and potential anti-tumor properties in both in vitro cellular models and in vivo animal studies [108]. The anti-tumor mechanism of anthocyanins primarily involves modulating cancer cell proliferation, autophagy, differentiation, and apoptosis through regulation of key signaling pathways, including tumor protein p53 , phosphatidylinositol 3-kinase/protein kinase (PI3K/Akt), and mitogen-activated protein kinase (MAPK) [109]. LRAs exhibited dose-dependent inhibition of human hepatocellular carcinoma (HepG2) cells, suppressing their proliferation, invasion, and migration capacity. Furthermore, an LRA induced G2 phase/mitosis phase (G2/M) cell cycle arrest, thereby promoting cellular apoptosis. It could also activate the adenosine monophosphate-activated protein kinase/mechanistic target of rapamycin (AMPK/mTOR) signaling pathway to inhibit autophagy in HepG2 cells, exhibiting excellent potential for use in the treatment of liver cancer [73]. Due to their synergistic interaction, the combined administration of LRAs and polysaccharides could inhibit human colon cancer cells by activating the PI3K/Akt and Janus kinase 2/signal transducer and activator of transcription 3 (JAK2/STAT3) pathways, thereby arresting the cell cycle and inducing apoptosis [74]. In addition, RAs have been shown to downregulate the expression of sirtuin 1(SIRT1) and upregulate the expression of the male absent on the first (MOF) and e1a-binding protein p300 (EP300) proteins, thereby affecting the acetylation levels of h4 (histone) and nuclear factor-κB (NF-κB) signaling pathway-related proteins (non-histone) in colorectal cancer, demonstrating their anti-colorectal cancer potential [75]. Another experiment showed that RAs can inhibit the activation of activator protein-1 (AP-1) and NF-κB and downregulate the expression of cyclooxygenase-2 (COX-2) and TNF by suppressing the expression of key transcription factors and proteins, thereby inhibiting the proliferation, invasion, metastasis, and angiogenesis of tumor cells [76]. In addition, RAs can block MAPK signaling pathway; inhibit the phosphorylation of p38 mitogen-activated protein kinase (p38), extracellular signal-regulated kinase (ERK), and c-jun n-terminal kinase (JNK); and block the activation of its upstream regulator mitogen-activated protein kinase kinase 4 (MKK4), thus inhibiting the malignant transformation and proliferation of tumor cells [76]. In summary, current experimental evidence clearly establishes the anti-cancer potential of anthocyanins. However, critical issues must be resolved to advance their application in cancer prevention. For example, the dose of anthocyanins required to trigger effects in vitro (10^−6^ to 10^−4^ M) far exceeds the amount observed in human plasma in vivo (10^−8^ to 10^−7^ M) due to limitations in anthocyanin bioavailability [108] (Figure 5). Current methods reported in the literature for improving bioavailability include nanodelivery systems, microencapsulation technology, formation of composite carriers with macromolecules, and chemical structural modifications [110,111,112,113]. Meanwhile, investigations of dose relationships and more human clinical trials are also important and necessary. Based on the results of research on the anticancer activity of anthocyanins in vitro and in vivo, future studies on the anticancer activity of anthocyanins should focus on bioavailability, drug formulation modification, and clinical data.

### 5.3. Anti-Aging Activity

With the increase in human life expectancy, age-related health conditions have emerged as significant public health concerns. Growing evidence indicates that anthocyanins exhibit potential anti-aging properties and may alleviate age-related diseases [114]. Cellular senescence is considered the main cause of aging and related diseases. Emerging evidence indicates that LRAs can alleviate UV-B-induced cellular damage by inhibiting apoptosis of fibroblasts, decreasing the expression of tumor necrosis factor-α (TNF-α) and caspase-7, and promoting the survival of heat shock factor (HSF) [77]. In addition, LRAs may reduce the accumulation of advanced glycation end products (AGEs) and malondialdehyde (MDA) in the serum of D-galactose-induced aging rats, increase the concentrations of key antioxidative biomarkers (including metallothionein (MT) and GSH) and antioxidant enzymes (including GSH-Px, CAT, and T-SOD), and reduce endogenous ROS. Thus, LRAs play an important role in the promotion of health in senescent rats [79]. In addition, it was found that the monomer compound of petunidin-3-O-(trans-p-coumaroylrutinoside)-5-O-glucoside isolated from LRAs could also alleviate neuroinflammation, oxidative stress, and liver and kidney damage in aging mice, exhibiting their anti-aging potential in vivo [80]. Research has demonstrated that RAs could inhibit the expression of matrix metalloproteinases by suppressing the MAPK/AP-1 and NF-κB pathways, activating the transforming growth factor-β/smad (TGF-β/Smad) pathway, and accelerating the synthesis of type I procollagen, showing good potential for preventing UV-induced skin aging [78]. These studies confirm that anthocyanins have considerable potential for preventing aging. The anti-aging mechanism of anthocyanins is not limited to a single pathway; it acts simultaneously on multiple key biological processes, such as antioxidant defense, inflammatory responses, cell survival and apoptosis, and extracellular matrix metabolism. This multi-target characteristic makes it a promising means of systematically intervening in the complex aging process.

### 5.4. Hypoglycemic Activity

Diabetes is a chronic metabolic disease characterized by impaired insulin secretion from pancreatic β-cells or insulin resistance (IR) of body. Diabetes can be classified into two main types: type 1 diabetes mellitus (T1DM) and type 2 diabetes mellitus (T2DM). T1DM occurs due to an innate lack of insulin in the body [115]. This primary defect necessitates therapeutic intervention through exogenous insulin replacement therapy. T2DM is primarily caused by unhealthy dietary habits, insulin resistance, and pancreatic islet β-cell dysfunction. At present, the main treatment methods are the consumption of a reasonable diet and use of hypoglycemic drugs. Studies have demonstrated that anthocyanins can exert hypoglycemic effects by improving insulin resistance, protecting β-cells, increasing insulin secretion, and reducing sugar digestion in the small intestine [116]. α-Glucosidase is a key enzyme involved in carbohydrate metabolism. It converts dietary carbohydrates into absorbable monosaccharides by hydrolyzing α-glycosidic bonds [81]. Experimental studies demonstrate that LRAs exhibit potent α-glucosidase inhibitory activity in Caco-2 cells, with an IC_50_ value of 25.3 μg/mL. Furthermore, LRAs significantly enhance glucose metabolism in IR HepG2 cells through activation of the PI3K/Akt signaling pathway and its downstream targets, glycogen synthase kinase 3β (GSK3β) and forkhead box O1 (FOXO1), resulting in an approximately 1.8-fold increase in the protein p-Akt/Akt ratio [81]. In the glycosidase inhibition experiment, the extraction of NTAs showed a 14-fold higher inhibition rate than that of acarbose, making them a potential hypoglycemic agent [82]. Furthermore, LRA supplementation has been demonstrated to improve hepatic gluconeogenesis and IR in mice with high-fat diet-induced obesity, which was achieved by decreasing the protein expression of toll-like receptor 4 (TLR4)/NF-κB/JNK signal pathway to ameliorate inflammation and activating the Nrf2/HO-1/(NQO1) NAD(P)H: quinone oxidoreductase 1 pathway to reduce oxidative stress in hepatic tissues [83]. Another study demonstrated that administering high doses of LRAs significantly reduced postprandial blood glucose in streptozotocin (STZ)-induced diabetic mice and was superior to the control drug acarbose [84]. Overall, although anthocyanins have demonstrated potential hypoglycemic activity at both the cellular and animal levels. They can exert hypoglycemic effects through various mechanisms, such as inhibiting intestinal glucose absorption, improving insulin resistance, protecting pancreatic beta cells, and regulating liver glucose metabolism. The research data are very encouraging and provide strong preclinical evidence for the development of anthocyanins as a new and efficient natural source for hypoglycemic functional food or drugs. However, more research on anthocyanin purity, absorption characteristics, and the dose–effect relationship in vivo and human clinical data regarding their effects is required.

### 5.5. Anti-Inflammatory Activity

Inflammation is a normal physiological response, but repeated stimulation or improper regulation can lead to chronic inflammation and a series of diseases [117]. Inflammation is regulated by a variety of factors, and the NF-κB signaling pathway is the main pathway of inflammation. Anthocyanins demonstrate significant anti-inflammatory potential by reducing the expression of TLR4 protein and suppressing the activation of the NF-κB and MAPK signaling pathways to inhibit the release of pro-inflammatory factors [118]. Experimental evidence has proven that LRAs can regulate inflammatory signaling pathways such as NF-κB and MAPK, inhibit the production and release of inflammatory cytokine TNF-α, interleukin-1β (Il-1β), and interleukin-6 (IL-6), and reduce the expression of inflammatory mediators, thereby attenuating the joint inflammatory response [87]. LRAs showed anti-inflammatory effects in a mouse model of dextran sodium sulfate (DSS)-induced colitis by blocking pro-inflammatory cytokines (TNF-α, IL-6, IL-1β, and Interferon-γ (IFN-γ)), increasing tight junction proteins (zonula occludens-1 (ZO-1), occludin, and claudin-1), and regulating intestinal flora [88]. In addition, NTAs dose-dependently decreased the release of the pro-inflammatory factors nitric oxide (NO), IL-1β, and TNF-α in RAW264.7 cells stimulated by lipopolysaccharide (LPS) [51]. Scientific evidence shows that RAs demonstrate excellent anti-inflammatory potential through the inhibition of two key inflammatory enzymes: lipoxygenase (LOX) and COX-2 [86,119]. Furthermore, the cyanidin-3-O-glucoside isolated from RAs has been shown to suppress NO production and exhibit anti-inflammatory activity. This mechanism is related to the inhibition of the protein expression of NF-κB and the activation of the MAPK signaling pathway [85]. Based on the above literature review, anthocyanins from all three berry plants exhibit potential anti-inflammatory activity. They can exert significant anti-inflammatory effects by inhibiting core inflammatory pathways and key enzyme expression, reducing the release of inflammatory factors, protecting tissue barriers, and regulating gut microbiota. They can target the “master switch” of inflammatory signaling networks, such as NF-κ B and MAPK, and systematically inhibit the occurrence and development of inflammation.

### 5.6. Neuroprotective Activity

With the increase in global life expectancy, Alzheimer’s disease (AD) has emerged as a significant public health challenge that substantially impacts quality of life in aging populations. Amyloid β-protein (Aβ) deposition, tau protein phosphorylation, and neurofibrillary tangles are the main pathological features of AD [120]. Experimental studies have demonstrated that anthocyanins, as natural substances found in plants, can reverse the protein expression of mitochondrial apoptotic pathway (bcl-2-associated x protein (Bax), cytochrome C, cysteine-dependent aspartate-directed protease-9 (caspase-9), and cysteine-dependent aspartate-directed protease-3 (caspase-3) and AD markers (Aβ, amyloid precursor protein (APP), phosphorylated tau (p-Tau), and β-secretase 1 (BACE-1)) induced by Aβ [121,122]. Experimental data demonstrated that LRAs significantly attenuated D-galactose-induced memory impairment and neuroinflammation in an AD mouse model. This neuroprotective effect was mediated through inhibition of the receptors for advanced glycation end products (RAGE)/NF-κB/JNK signaling pathway [50]. Furthermore, RAs could prevent inflammation-related neurodegenerative diseases by downregulating the NADPH oxidase 2–thioredoxin-interacting protein–nod-like receptor pyrin domain-containing protein 3 signaling axis, decreasing ROS production and inhibiting the secretion of inflammatory factors (interleukin-18 (IL-18) and interleukin-1 (IL-1)) in BV2 microglia in the brain [89]. It was proven that RAs can inhibit Aβ fibrillization and reduce inflammation and elevated ROS induced by LPS in BV2 microglial cells. These studies suggested that RAs have potential neuroprotective effects [90]. In addition, NTAs significantly downregulated the hippocampal protein expression of both RAGEs and Aβ in a D-galactose-induced AD rat model. Concurrently, they suppressed glial cell overactivation, demonstrating potent neuroprotective efficacy [53]. In summary, anthocyanins exert anti-AD effects by intervening in pathways such as Aβ deposition, tau protein phosphorylation, neuroinflammation, and oxidative stress. Anthocyanins or their active metabolites must be able to effectively cross the blood–brain barrier and reach a concentration in the brain sufficient to exert pharmacological effects. Future research needs to clarify its ability and form of crossing the blood–brain barrier. The value of anthocyanins lies in their potential as a long-term dietary supplement to prevent or delay the occurrence of disease.

### 5.7. Impacts on Gut Microbiota

The homeostasis of gut microbiota has been demonstrated to play a pivotal role in maintaining human health and is closely associated with the pathogenesis of numerous diseases. Consequently, the gut microbiome has been regarded as the “second genome” in human physiology [123]. Anthocyanins can promote the growth of beneficial bacteria and inhibit harmful bacteria to regulate the intestinal microbiota [124]. For example, LRAs exert a positive effect on the intestinal flora by increasing the relative abundances of the beneficial bacteria *Bifidobacterium* and *Allisonella*, reducing the relative abundances of the harmful bacteria *Prevotella*, *Dialister*, *Megamonas*, and *Clostridium*, and significantly decreasing the ratio of thick-walled bacteria and *Anthrobacterium* bacteria (from 0.57 to 0.28), as well as producing short-chain fatty acids (SCFAs) through intestinal microbiota [91]. Moreover, LRAs can alter the structure of gut microbiota in high-fat-diet model mice, thereby preventing obesity. LRAs achieved this effect by decreasing the relative abundance of *Firmicutes*, *Lactobacillaceae*, *Streptococcaceae*, and *Erysipelotrichaceae* in the intestinal flora and increasing the mRNA expression levels of ZO-1 and claudin in the colons of high-fat-diet mice, thereby enhancing their intestinal barrier function [93]. Studies have demonstrated that 2 weeks of continuous intervention with 200 mg/kg/day of an LRA not only increased the number of large and small intestinal goblet cells and significantly increased the mRNA expression of intestinal barrier proteins (ZO-1, occludin, claudin-1, and mucin 1) to promote intestinal integrity but also increased the abundance of *Barnesiella, Alistipes*, *Eisenbergiella*, *Coprobacter*, and *Odoribacter* to regulate the intestinal flora and maintain intestinal health [72]. In addition, although LRAs are poorly absorbed, they can be degraded to produce metabolites such as phenolic acids and SCFAs, which may play a homeostatic role in the intestinal environment by lowering the intestinal pH, inhibiting the propagation of pathogenic bacteria, and enhancing intestinal barrier function [92]. RAs have been proven to alter the intestinal flora and enhance its integrity by increasing the abundance of *Prevotella* and the ratio between *Bacteroidetes* and *Firmicutes* [94]. The regulatory effect of anthocyanins on gut microbiota is a key bridge connecting their low bioavailability and broad health benefits. Treating gut health as a core target provides a new and more solid foundation for understanding and developing the application of anthocyanins in the prevention and treatment of metabolic, inflammatory, and neurodegenerative diseases.

### 5.8. Other Biological Activities

As naturally occurring pigments with diverse pharmacological properties, anthocyanins have garnered significant scientific interest due to their multifaceted biological activities. Apart from the biological activities mentioned above, anthocyanins also display anti-fatigue, antibacterial, anti-cardiovascular, and retina-protective effects. Studies have shown that LRAs may be able to effectively improve fatigue status, demonstrating that they can increase exercise endurance in fatigued rodents, increase glucose reserves (Glu, liver glycogen, and muscle glycogen), scavenge free radicals, and improve metabolism (blood urea nitrogen (BUN) and cortisol) [98]. LRAs have demonstrated broad-spectrum antimicrobial activity against multiple pathogens, such as *Staphylococcus aureus*, *Escherichia coli*, *Aspergillus niger*, and *Penicillium* spp. Their antibacterial mechanism is related to their ability to disrupt cell membrane integrity [95]. Furthermore, RAs displayed notable growth-inhibiting effects against *Bacillus cereus*, *Corynebacterium diphtheriae*, and *Moraxella catarrhalis* [96,97]. In addition, due to LRAs’ excellent antioxidant and anti-inflammatory pharmacological activities, they exhibited a protective effect against retinal damage induced by blue light irradiation. This mechanism is related to the activation of the Nrf2/HO-1 pathway, reducing ROS, and the ability to attenuate NF-κB-induced inflammation and apoptosis induced by the activation of the Bcl2/Bax signaling pathway to upregulate endogenous antioxidant enzymes [99]. Moreover, RAs can also protect retinal pigment epithelial cells from apoptosis by reducing the activities of protein tyrosine phosphatase 1B (PTP1B) and caspase-1 and inhibiting axial PTP1B-ERS, suggesting their potential therapeutic value for diabetic retinopathy [100]. In addition, RAs were demonstrated to exhibit a significant preventive effect against alcoholic liver disease. This effect is attributed to their ability to reduce ROS induced by hepatic histopathological changes and decrease apoptosis via the PI3K-Akt signaling pathway [101]. In addition, NTAs showed great potential in the prevention of cardiovascular diseases by limiting ROS generation, promoting the activity of key antioxidant enzymes, enhancing the glutathione redox cycle, influencing apoptotic signaling alterations, and ultimately mediating the caspase-dependent cell death pathway [52]. The above research highlights the enormous potential of anthocyanins as a natural multifunctional health ingredient, not only providing potential natural strategies for preventing various chronic diseases but also opening up a path for development in the fields of nutritional supplements and medicine.

## 6. Applications in Food Industry

Within the food sector, anthocyanins are commonly utilized as natural colorants, packaging materials and smart labels. The diverse bioactive properties of anthocyanins establish their potential as functional food ingredients and considerably increase market demand for anthocyanins in health supplements.

### 6.1. Natural Colorants

Growing consumer demand for healthier and safer food products has driven increased research into natural alternatives to synthetic additives. Potential safety issues associated with artificial colorants have intensified the search for plant-derived substitutes. The EFSA has approved the use of anthocyanins as food dyes and acknowledged their safety for consumption in food products. For instance, LRA extracts not only impart desirable color to yogurts and fermented dairy products but also enhance their nutritional profile due to their strong antioxidant activity, providing potential health benefits to consumers. It should be emphasized that LRAs are a more effective natural purple colorant than purple sweet potato extract in yogurt and fermented milk formulations [125]. Recent studies have shown that acylated NTA extracts produced through macroporous resin purification offer a viable solution for the large-scale production of stable natural pigments [22]. RA extract has been demonstrated to possess considerable potential as a food additive, owing to its favorable antioxidant properties and demonstrable antibacterial effects against food-associated microorganisms [126]. However, the use of anthocyanins as food colorants is associated with several limitations. Structural instability leads to rapid degradation, posing challenges for industrial-scale application [127]. Additionally, anthocyanins are generally more costly than synthetic alternatives. To address these issues, various strategies have been developed to improve anthocyanin stability, such as microencapsulation and structural modification [128].

### 6.2. Packaging Materials and Smart Labels

Anthocyanins have been widely utilized in the development of edible packaging films. Their key advantage lies in offering a more environmentally friendly and healthier alternative to conventional packaging materials while also enabling real-time monitoring of food freshness to alleviate consumer concerns regarding food safety [129]. Nano-encapsulated LRAs were incorporated into a starch/polyvinyl alcohol matrix to fabricate smart packaging films. The resulting films exhibited a dense microstructure, strong intermolecular interactions, high water vapor barrier capacity, and excellent mechanical properties, which could effectively extend the freshness of refrigerated largemouth bass (*Micropterus salmoides*) fillets [130]. In another application, LRAs were integrated into a film composed of soy protein isolate, xanthan gum, and agar and applied to extend lamb shelf life [131]. Furthermore, LRAs exhibit sensitivity to changes in meat freshness, specifically by reacting to ammonia gas and undergoing reactive discoloration in response to pH variations [33]. Based on these characteristics, LRAs could be immobilized onto porous filter paper to prepare LRA paper smart labels to provide real-time and precise feedback on fish freshness, thereby safeguarding consumer health [33]. Furthermore, LRA was used to prepare a novel pH-sensitive membrane gel that serves as an indicator for monitoring meat freshness during storage [132].

### 6.3. Functional Food and Health Supplements

Thanks to their broad spectrum of activity, anthocyanins have captured consumer attention and gained market prominence. Research has demonstrated that RA extracts are effective natural antioxidant preservatives, extending product shelf life by scavenging free radicals and suppressing oxidative degradation in food matrices [133]. LRA extract was added to yogurt and fermented milk formulations to provide nutrition and health benefits [125]. Lyophilized LRA extract powders could effectively neutralize free radicals in the body to slow the aging process, enhance sleep quality, and boost immune function [134]. In addition, LRA extract exhibits significant skin-enhancing properties and has been used as a functional additive in cosmetics to promote whitening effects [134]. Thus, these anthocyanin extracts display excellent potential as key ingredients in health foods, cosmetics, and therapeutic products.

If anthocyanins are used in food, the safe dosage should be clarified. Since anthocyanins are not considered nutritionally essential, most dietary guidelines do not specify the recommended daily intake of anthocyanins. According to the European Food Safety Authority, available toxicological data remain insufficient to establish a definitive daily intake value [135]. In contrast, the Chinese Nutrition Society’s Dietary Reference Intakes recommend a daily consumption of 50 mg [13]. Moreover, the Joint FAO/WHO Expert Committee on Food Additives has set an Acceptable Daily Intake for anthocyanins at 2.5 mg/kg body weight per day [136]. Children, pregnant women, and individuals with impaired liver or kidney function should reduce their dosage by 20–30% based on their physiological condition to ensure safe consumption.

In summary, the application of anthocyanins in the food industry perfectly embodies the modern interpretation of “medicinal food homology”. Anthocyanins not only provide “color” but are an innovative component that aids in preservation, food safety monitoring, and health promotion, representing an important direction for the future development of the food industry. However, there is not yet a global standard for the safe intake of anthocyanins. More toxicology research is urgently needed to establish clearer and more unified regulatory standards.

## 7. Limitations and Future Prospects

Several challenges remain in the development and utilization of anthocyanins among all plants in nature, which require further investigation and clarification to maximize their potential benefits for human health. The key issues include the following: (1) Biosynthesis of anthocyanins—While the anthocyanin biosynthetic pathway has been well characterized, its practical application and large-scale production remain constrained by the pathway’s complexity and the low efficiency of heterologous synthesis. To date, only cyanidin-3-O-glucoside has been successfully synthesized in microbial systems [137]. Future efforts to enhance anthocyanin production may focus on two key strategies: optimizing plant host biosynthesis pathways through targeted overexpression and developing efficient microbial cell factories for heterologous production. (2) Stability of anthocyanins—The inherent structural instability of anthocyanins, dictated by their molecular properties, significantly hinders their broader application and commercial potential. Future research should focus on developing protective technologies such as microencapsulation, nanocarrier systems, and liposome embedding to enhance anthocyanin stability [138,139]. These advancements will facilitate the creation of high-value anthocyanin products and drive the sustainable growth of the anthocyanin industry. (3) Mechanistic target of anthocyanin—While current pharmacological research has explored anthocyanin activity both in vivo and in vitro at the protein and mRNA levels, critical gaps remain in understanding their metabolic pathways and specific pharmacodynamic targets. Future studies should employ an integrated approach combining metabolomics, proteomics, gene knockdown techniques, and target fishing strategies across diverse cell types and animal models to comprehensively elucidate anthocyanins’ metabolic pathways and molecular targets. (4) Bioavailability and clinical efficacy of anthocyanins—Current research on anthocyanin efficacy primarily relies on cellular and animal models, with a notable paucity of clinical data from human studies [140]. Furthermore, consumer acceptance of anthocyanins derived from these three berry species remains limited compared to more established sources like blueberry and purple potato products.

In the future, clinical trials on anthocyanins should be strengthened, with a particular emphasis on large-scale population-based studies to comprehensively evaluate their therapeutic potential against various diseases. Further research is needed to elucidate their absorption, distribution, metabolism, and excretion through detailed pharmacokinetic investigations. It would also be valuable to explore the synergistic effects of anthocyanins with other bioactive compounds, as well as their long-term benefits and safety profiles across diverse demographic groups. Additionally, utilizing advanced biomarkers and omics technologies could provide deeper insights into their mechanisms of action. The outcomes of these studies could better support human health and improve quality of life.

## 8. Conclusions

*Lycium ruthenicum* Murr., *Nitraria tangutorun* Bobr, and *Rubus idaeus*, well-known medical and edible berry plants grown on the Qinghai–Tibet Plateau, are rich in anthocyanins. This study is a multifaceted review encompassing the structural characterization, biosynthetic pathway, pharmacological properties, and food industry applications of anthocyanins. The anthocyanin structure exhibits diversity regarding the presence of glycosylation and acylation. These compounds demonstrate beneficial effects both in vitro and in vivo and have diverse potential applications in the food industry, where they can used as natural colorants, in packaging materials, and in smart labels; as functional foods; and in health supplements. However, more clinical research data on their effectiveness and safety, as well as extraction mechanisms, are needed for their development and application.

## Figures and Tables

**Table 1:** Anthocyanins from different sources.

Sources	Main Anthocyanin Types	Total Anthocyanins Content (mg C3G/100 g FW)	References
*Lycium ruthenicum* Murr.	Petunidin-3-*O*-(trans-p-coumaroyl-rutino side)-5-glucoside Pentunidin-3-*O*-(*cis*-*p*-coumaroyl-rutinoside)-5-*O*-glucoside Petundin-3-*O*-galactoside-5-*O*-glucoside Petunidin-3-*O*-(caffeoyl-rutinoside)-5-*O*-glucoside	450–550	[21]
*Nitraria tangutorun*	Cyanidin-3-*O*-(*trans*-*p*-coumaroyl)-diglucoside Cyanidin-3-*O*-(*cis-p*-coumaroyl)-diglucoside Cyanidin-3-*O*-(caffeoyl)-diglucoside	8.1–239.86	[22,23]
Raspberry	Cyanidin-3-*O*-glucoside Cyanidin-3-*O*-rutinoside Cyanidin-3-*O*-sophoroside Cyanidin-3-*O*-sambubioside Cyanidin-3-*O*-xylosylrutinoside	14.7–336	[24,25]
Blueberry	Cyanidin-3-*O*-galactoside Petunidin-3-*O*-glucoside Peonidin-3-*O*-galactoside Delphinidin-3-*O*-galactoside Delphinidin-3-*O*-glucoside	69.97–378.31	[26,27]
Strawberry	Pelargonium-3-*O*-rutinoside Cyanidin-3-*O*-glucoside Pelargonium-3-*O*-glucoside	29.00–49.43	[27,28]
Blackcurrant Cultivars	Delphinidin-3-*O*-glucoside Cyanidin-3-*O*-rutinoside Delphinidin 3-*O*-rutinoside Cyanidin-3-*O*-utinoside	62.80–271.33	[27,29]
Cranberry	Cyanidin-3-*O*-galactoside Cyanidin-3-*O*-arabinoside Peonidin-3-*O*-galactoside Peonidin-3-*O*-arabinoside	11.10–32.00	[27,30]
Sweet cherry	Cyanidin-3-*O*-glucoside Cyanidin-3-*O*-rutinoside Peonidin-3-*O*-rutinoside Pelargonidin-3-*O*-rutinoside	1.03–179.14	[27,31]

**Table 2:** The extracted methods for anthocyanins from three berry plants.

Sources	Extraction Method	Optimal Extraction Condition	Extraction Yield	References
*Lycium ruthenicum* Murr.	Semi-continuous liquid phase pulsed electrical discharge system	Solvent composition: 20% ethanol Input voltage: 8 KV Extraction time: 8 min Solid–liquid ratio: 1:2 g/mL	15.75 ± 0.28 mg/g DW	[35]
	Ultrasonic microwave synergistic extraction	Solvent composition: 1% HCl-70% ethanol Extraction time: 26.141 min Ultrasonic power: 216.253 W Microwave power: 89.311 W Solid–liquid ratio: 1:17.294 g/mL	10.157 mg/g DW	[36]
	Enzyme-assisted extraction	Solvent composition: 0.1%HCl water-containing pectinase Extraction temperature: 37 min Extraction time: 38 °C Solid–liquid ratio: 1:20 g/mL	19.51 ± 0.21 mg/g DW	[37]
	Ultrasonic extraction	Solvent composition: 90% ethanol Extraction temperature: 40 °C Ultrasonic power: 300 W Extraction time: 30 min Solid–liquid ratio: 1:20 g/mL	27.66 mg/g DW	[8]
	Ultrasonic-assisted enzymolysis extraction	Solvent composition: 0.24% pectinase Extraction temperature: 48 °C Extraction time: 21 min Solid–liquid ratio: 1:21 g/mL	31.6 mg/g DW	[34]
*Nitraria tangutorun*	Aqueous two-phase extraction	Solvent composition: 28% NaH_2_PO_4_ Extraction temperature: 65 °C Extraction time: 45 min Ultrasonic power: 300 W Solid–liquid ratio: 1:10 g/mL	3.62 ± 0.05 mg/g DW	[38]
	Ultrasound-assisted extraction	Solvent composition: 54% ethanol containing 0.1%HCl Extraction temperature: 68 °C Extraction time: 30 min Ultrasonic power: 300 W Solid–liquid ratio: 15 g/mL	3.862 mg/g DW	[39]
	Ultrasound-assisted deep eutectic solvent extraction	Solvent composition: Choline chloride/1,2-propanediol containing 25% water Extraction temperature: 50 °C Extraction time: 30 min Ultrasonic power: 300 W	1.413 ± 0.054 mg/g DW	[40]
Raspberry	Subcritical water extraction	Solvent composition: superheated water Extraction temperature: 130 °C Flow rate: 3 mL/min Extraction time: 9 min Extraction pressure: 7 MPa	0.98 ± 0.33 mg/g FW	[41]
	Ultrasound-assisted deep eutectic solvent extraction	Solvent composition: 1,4-butanediol as the HBD and mole ratio of 1:3 Extraction temperature: 51 °C Extraction time: 32 min Ultrasonic power: 210 W	1.378 mg/g DW	[42]
	Ultrasound-assisted extraction	Solvent composition: 1.5 M HCl–95% ethanol (15:85) Extraction temperature: 51 °C Extraction time: 200 s Ultrasonic power: 400 W Solid–liquid ratio: 1:4 g/mL	0.345 mg/g FW	[24]
	Microwave-assisted extraction	Solvent composition: 52% ethanol Extraction temperature: 55 °C Extraction time: 4 min Microwave power: 469 W Solid–liquid ratio: 1:25 g/mL	2.18 ± 0.06 mg/g DW	[43]

Notes: DW, dry weight. FW, fresh weight.

**Table 3:** Anthocyanins from *Lycium ruthenicum* Murr. (LRM) fruit.

NO.	Compound Name	Position 3	Position 5	Analytical Method	References
Petunidin					
1	Petundin-3-O-galactoside-5-O-glucoside	Gal	Glu	HPLC-MS	[21]
2	Petundin-3-O-glucoside-5-O-glucoside	Glu	Glu	HPLC-MS/NMR	[21]
3	Petunidin-3-O-rutinoside (caffeoyl)-5-O-glucoside	Rut (caffeoyl)	Glu	HPLC-MS	[21]
4	Pentunidin-3-O-rutinoside (cis-p-coumaroyl)-5-O-glucoside	Rut (cis-p-coumaroyl)	Glu	HPLC-MS/NMR	[21]
5	Pentunidin-3-O-rutinoside (trans-p-coumaroyl)-5-O-glucoside	Rut (trans-p-coumaroyl)	Glu	HPLC-MS	[21]
6	Pentunidin-3-O-glucoside (maloyl)-5-O-glucoside	Glu (maloyl)	Glu	HPLC-MS	[21]
7	Pentunidin-3-O-glucoside (feruloyl)-5-O-glucoside	Glu (feruloyl)	Glu	HPLC-MS	[21]
8	Petunidin-3-O- rutinoside isomer-(p-coumaroyl)	Rut isomer-(p-coumaroyl)	OH	HPLC-MS	[37]
Delphinidin					
9	Delphinidin-3-O-rutinoside (cis-p-coumaroyl)-5-O-glucoside	Rut (cis-p-coumaroyl)	Glu	HPLC-MS	[21]
10	Delphinidin-3-O-rutinoside (trans-p-coumaroyl)-5-O-glucoside	Rut (trans-p-coumaroyl)	Glu	HPLC-MS	[21]
11	Delphinidin-3-O-rutinoside-5-O-glucoside	Rut	Glu	HPLC-MS	[37]
12	Delphinidin-3-O-(p-coumaroyl)-glucoside	(p-coumaroyl)-Glu	OH	HPLC-MS	[37]
Malvidin					
13	Malvidin-3-O-rutinoside (cis-p-coumaroyl)-5-O-glucoside	Rut (cis-p-coumaroyl)	Glu	HPLC-MS	[21]
14	Malvidin-3-O-rutinoside(trans-p-coumaroyl)-5-O-glucoside	Rut (trans-p-coumaroyl)	Glu	HPLC-QTOF-MS/MS	[48]
15	Malvidin-3-O-rutinoside(feruloyl)-5-O-glucoside	Rut (feruloyl)	Glu	HPLC-QTOF-MS/MS	[48]
16	Malvidin-3-O-rutinoside-5-O-glucoside	Rut	Glu	HPLC- QTOF-MS/MS	[48]
Pelargonidin					
17	Pelargonidin-3-O-galactoside	Gal	OH	HPLC-MS	[49]
18	Pelargonidin-3-O-diglucoside	Di-Glu	OH	HPLC-MS	[49]
19	Pelargonidin-3-O-glucoside	Glu	OH	HPLC-MS	[49]
Cyanidin					
20	Cyanidin-3-O-galactoside	Gal	OH	UPLC- TOF/MS	[50]
21	Cyanidin-3,5-O-diglucoside	Di-Glu	Di-Glu	UPLC- TOF/MS	[50]
22	Cyanidin-3-O-glucoside	Glu	OH	UPLC- TOF/MS	[50]

Note: Glu, glucoside; Rut, rutinoside; Di-Glu, diglucoside; Gal, galactoside.

**Table 4:** Anthocyanins from NTB fruit.

NO.	Compound Name	Position 3	Position 5	Analytical Method	References
	Cyanidin				
1	Cyanidin-3-O-diglucoside-isomer	Di-Glu-isomer	OH	UPLC-MS	[51]
2	Cyanidin-3-O-hexose	hexose	OH	UPLC-MS	[51]
3	Cyanidin-3-O-(feruloyl)-diglucoside	(feruloyl)-Di-Glu	OH	UPLC-MS	[51]
4	Cyanidin-3-O-(cis-p-coumaroyl)-diglucoside	(cis-p-coumaroyl)- Di-Glu	OH	UPLC-MS	[51]
5	Cyanidin-3-O-(trans-p-coumaroyl)-diglucoside	(trans-p-coumaroyl)-Di-Glu	OH	UPLC-MS/NMR	[51]
6	Cyanidin-3-O-(p-coumaroyl)-glucoside	(p-coumaroyl)-Glu	OH	UPLC-MS	[51]
7	Cyanidin-3-O-diglucoside	Di-Glu	OH	HPLC-ESI-MS	[52]
8	Cyanidin-3-O-sambubioside	sambubioside	OH	HPLC-ESI-MS	[52]
9	Cyanidin-3-O-(cis-caffeoyl)- diglucoside	(cis-caffeoyl)-Di-Glu	OH	UPLC- TOF-MS	[53]
10	Cyanidin-3-O-(trans-caffeoyl)- diglucoside	(trans-caffeoyl)-Di-Glu	OH	UPLC-TOF-MS	[53]
	Malvidin				
11	Malvidin-3-O-glucoside	Glu	OH	HPLC-ESI-MS	[52]
12	Malvidin-3-O-(acetyl)-glucoside	(acetyl)-Glu	OH	HPLC-ESI-MS	[52]
13	Malvidin-3-O-(coumaroyl)-glucoside-5-O-glucoside	(coumaroyl)-Glu	Glu	HPLC-ESI-MS	[52]
14	Malvidin-3-O-(cis-coumaroyl)-glucoside	(cis-coumaroyl)-Glu	OH	HPLC-ESI-MS	[52]
15	Malvidin-3-O-(trans-coumaroyl)-glucoside	(trans-coumaroyl)-Glu	OH	HPLC-ESI-MS	[52]
	Peonidin				
16	Peonidin-3-O-diglucoside	Di-Glu	OH	UPLC-MS	[51]
17	Peonidin-3-O-(coumaroyl)-glucoside-5-O-glucoside	(coumaroyl)-Glu	Glu	HPLC-ESI-MS	[52]
18	Peonidin-3-O-(coumaroyl)-glucoside	(coumaroyl)-Glu	OH	HPLC-ESI-MS	[52]
	Pelargonidin				
19	Pelargonidin-3-O-diglucoside-isomer	Di-Glu-isomer	OH	HPLC-ESI-MS	[52]
20	Pelargonidin-3-O-(caffeoyl)-diglucoside	(caffeoyl)-Di-Glu	OH	HPLC-ESI-MS	[52]
21	Pelargonidin-3-O-(coumaroyl)-diglucoside	(coumaroyl)-Di-Glu	OH	HPLC-ESI-MS/NMR	[52,54]
22	Pelargonidin-3-O(ferulyl)- diglucoside	(ferulyl)-Di-Glu	OH	UPLC-TOF-MS	[53]
	Delphinidin				
23	Delphinidin-3-O-(cis-p-coumaroyl)-glucoside-5-O-glucoside	(cis-p-coumaroyl)-Glu	Glu	UPLC-MS	[51]
24	Delphinidin-3-O-(trans-p-coumaroyl)-glucoside-5-O-glucoside	(trans-p-coumaroyl)-Glu	Glu	UPLC-MS	[51]
25	Delphinidin-3-O-(coumaroyl)-glucoside	(coumaroyl)-Glu	OH	HPLC-ESI-MS	[52]

Notes: Glu, glucoside; Di-Glu-diglucoside; Gal, galactoside.

**Table 5:** Anthocyanins from RI fruit.

NO.	Compound Name	Position 3	Position 5	Analytical Method	References
	Cyanidin				
1	Cyanidin-3-O-glucoside	Glu	OH	HPLC-ESI-MS	[58]
2	Cyanidin-3-O-sambubioside	sambubioside	OH	HPLC-ESI-MS	[58]
3	Cyanidin-3-O-xylosylrutinoside	xylosylrutinoside	OH	HPLC-ESI-MS	[58]
4	Cyanidin-3-O-rutinoside	Rut	OH	HPLC-ESI-MS/NMR	[58]
5	Cyanidin-3-O-sophoroside	sophoroside	OH	HPLC	[59]
6	Cyanidin-3-O- (2G-glucosyl-rutinoside)	Glucosyl- Rut	OH	HPLC	[59]
	Pelargonidin				
7	Pelargonidin-3-O-rutinoside	Rut	OH	HPLC-ESI-MS	[58]
8	Pelargonidin-3-O-sophoroside	sophoroside	OH	HPLC	[59]
9	Pelargonidin-3-(2G-glucosyl-rutinoside)	Gucosyl-Rut	OH	HPLC	[59]
10	Pelargonidin-3-glucoside	Glu	OH	HPLC	[59]

Notes: Glu, glucoside; Rut, rutinoside.

**Table 6:** Bioactivity of anthocyanin derived from three berry plants.

Benefits	Treatment	Evaluation Items	Reference
Antioxidant activity	In vitro studies		
	Petunidin monomer from LRM (10 μM) on CML-induced oxidative stress in Neuro-2a cells	↓ROS, ↓MDA, ↑GSH	[69]
	LRA extracts	↓DPPH, ↓·OH, ↓ABTS, ↓O_2_^•−^ and ↓ FRAP radicals	[36]
	Petunidin monomer from LRM (1–10 μM) on H_2_O_2_-induced neuron-like cells	↑CAT, ↑SOD, ↑GSH-Px, ↓MDA	[70]
	RA extracts (containing 6 anthocyanins)	Rate of DPPH and ABTS radical scavenging was 83.77% and 66.66%	[41]
	In vivo studies		
	Cyanidin monomer from NTB (50 mg/kg) was administrated once daily to D-galactose-induced rats for 7 weeks	↓ROS, ↓MDA, ↑T-SOD, ↑GSH	[53]
	The NTA extracts (0.35, 1.05 and 2.10 g/kg) for 4 weeks on hyperlipemia rats	↑TAC, ↑SOD, ↓MDA	[71]
	LRA extracts (200 mg/kg/d) on C57BL/6 mice for 12 weeks	↑T-AOC, ↑T-SOD, ↑CAT, ↑GSH, ↑GSH-Px, ↓AST, ↓ALT, ↓ALP and MDA↓	[72]
Anti-tumor activity	In vitro studies		
	Treatment of HepG2 cells with 500 and 1000 μg/mL LRA extracts for 24 h	↓Cell viability and proliferation, ↓migration and invasion, ↑AMPK/mTOR, ↓G2/M phase, ↑Beclin-1, p62 and LC3-II/LC3-I	[73]
	Combined treatment with LRA extracts (20 μg/mL) and polysaccharide extracts from LRM on LoVo cells for 24 h	↓Cell activity, ↓G0-G1 phase, ↑apoptosis, ↑PI3K-Akt, ↑p-JAK2 and p-STAT3, ↑Caspase-3, ↑Bax/Bcl-2, ↑ROS	[74]
	Applying RA extracts to AOM-induced colorectal cancer mice (4.1 g/kg) for 45 days and the CRC cell lines SW480 and Caco-2 cell (0, 25, 50 μg/mL)	↓Sirtuin1, ↑MOF and EP300, ↑acetylated-p65, ↑NF-κB, ↑Bax, ↓Bcl-2, ↓cyclin-D1, ↓cellular myelocytomatosis oncogene, ↓NLRP3	[75]
	Cyanidin-3-glucoside monomer from RAs on TPA-induced JB6 cells (10, 20, 40 μM)	↓MAPK, ↓transactivation of NF-κB and AP-1, ↓COX-2, ↓TNFα, ↓AP-1,	[76]
Anti-aging activity	In vitro studies		
	LRA extracts (0.1, 0.5, 1.0 mg/mL) against UVB-induced human skin fibroblast cells	↓Apoptosis rate of HSFs, ↓TNF-α, ↓caspase-7, ↑survivin	[77]
	RA extracts against UVB-induced NHDFs cell (1, 10, 100 μg/mL)	↓MMP, ↓MAPK, ↓NF-κβ, ↓IL-1β ↓AP-1, ↑Nrf2, ↑TGF-β/Smad	[78]
	In vivo studies		
	LRA extracts on D-galactose-induced aging rats (100 mg/kg/d, 8 weeks)	↓AGEs, ↓MDA, ↑metallothionein, ↓GSH, ↑GSH-Px, ↑CAT, ↑T-SOD, ↓ TNF-α, ↓IL-6, ↑IL-10	[79]
	Petunidin monomer from LRMs on D-galactose-induced aging mice (50, 100 mg/kg, 8 weeks)	↑SOD, ↑GSH, ↓MDA, ↓AChE, ↓Iba1, ↓GFAP, ↓BACE-1, ↓Aβ (1–42)	[80]
Hypoglycemic activity	In vitro studies		
	LRA extracts on Caco-2 cells (0, 5, 10, 20,40, 80 μg/mL) and IR-HepG2 (25 μg/mL)	IC_50_ value of α-glucosidase was 25.3 μg/mL, ↑glucose consumption and uptake, ↑PI3K/Akt, ↑GSK3β, ↑FOXO1, ↓ROS	[81]
	NTA extracts (containing 8 anthocyanins)	IC_50_ value of α-glucosidase was 0.1807 ± 0.0135 mg/mL	[82]
	In vivo studies		
	LRA extracts (50, 100, 200 mg/kg/d for 12 weeks) against high-fat diet-induced insulin resistance in mice	↓AUC, ↓HOMA-IR, ↓fasting insulin, ↓GHb, ↑IRS-1/AKT, ↑Nrf2/HO-1/NQO1, ↓TLR4/NF-κB/JNK	[83]
	LRA extracts (containing 10 anthocyanins) on α-glucosidase in vivo (200 and 400 mg/kg)	IC_50_ value of α-glucosidase was 4.468 mg/mL, ↓AUC, ↓PBG	[84]
Anti-inflammatory activity	In vitro studies		
	RA extracts on J774 cells (0, 11, 22, 45 and 90 μg/mL) by LPS stimulation	↓NO, ↓iNOS, ↓NF-κB, ↓ERK-1/2	[85]
	RA extracts	IC_50_ value of LOX was 4.85 mg FW/mL, IC_50_ value of COX-2 was 2.25 mg FW/mL	[86]
	In vivo studies		
	LRA extracts (200 mg/kg) and petunidin-3-glu (40 mg/kg) against gouty arthritis induced by monosodium urate	TNF-α, ↓IL-1β, ↓IL-18, ↓PE2, ↓COX-1, ↓paw COX-1 mRNA	[87]
	LRA extracts (200 mg/kg/d) and the main monomer (P3G) against DSS-induced colitis in mice	↑body weight, ↑solid fecal weight, ↑colon length, ↓DAI, ↓TNF-α, ↓IL-6, ↓IL-1β, ↓IFN-γ,↑ZO-1, ↑occludin, ↑claudin-1	[88]
Neuroprotective activity	In vitro studies		
	RA extracts against LPS-induced BV2 microglia (3, 10, 30, 100 μg/mL)	↓iNOS, ↓ROS, ↓gp91 phox, ↓NLRP3, ↓caspase-1, ↓TXNIP, ↑TRX	[89]
	RA extracts (20 μg/mL) on BV-2 Microglia	↓Aβ Fibrillation, ↓NOS, ↓ROS, ↓Caspase-3/7, ↓AGEs	[90]
	In vivo studies		
	LRA extracts on D-galactose-treated rats (50, 100, 200mg/kg for 7 weeks)	↓p-JNK, ↓caspase-3, ↓Bax/Bcl2, ↓ memory impairment, ↓RAGE	[50]
	NTA extracts on D-galactose-treated rats (50, 100mg/kg for 7 weeks)	↑learning and memory, ↓RAGE,↓GFAP, ↓Iba-1, ↓ROS, ↓Aβ, ↓gliosis in the hippocampus	[53]
Impacts on gut microbiota	In vitro studies		
	Effect on human intestinal microbiota of LRA extracts in vitro	↓*Firmicutes*, ↓*Bacteroidetes*, ↑*Actinobacteria*,↑*Bifidibacterium*, ↓ *Allisonella*,↓*Prevotella*, ↓*Dialister*, ↓*Megamonas*, ↓*Clostridium*, ↑*Allisonella*, ↑*Sutterellaceae*, ↑*Blautia*, ↓*Phascolarctobacterium*, ↓*Lachnospiraceae*, ↓*Faecalibacterium*	[91]
	Effect of LRA extracts and the main monomer on gut microbiota of feces from patients with inflammatory bowel disease	↑*Lactobacillus*, ↑*Bifidibacteria*, ↓*Escherichia/Shigella*, ↓SCFA, ↑*Prevotella*	[92]
	In vivo studies		
	The main LRA monomer on the HFD-induced obesity mice (100 mg/kg)	↓LPS, ↑Claudin, ↑ZO-1, ↓IL-6, ↓IL-1β, ↓Firmicutes, ↓*Lactobacillaceae,* ↓*Streptococcaceae*, ↓*Ruminococcaceae*, ↓*Erysipelotrichaceae*, ↑*Bifidobacteriaceae*, ↑*Helicobacteraceae*, ↑*Deferribacteraceae*	[93]
	Intake of LRA extracts of mice (200 mg/kg/d for 12 weeks)	↑ZO-1, ↑occludin, ↑claudin-1, ↑muc1, ↑*Barnesiella*, ↑*Alistipes*, ↑*Eisenbergiella,* ↑*Coprobacter*, ↑*Odoribacter*, ↑SCFA	[72]
	Pelargonidin-3-O-glucoside anthocyanin monomer from raspberry on db/db diabetic mice (150 mg/kg)	↑occludin, ↑ZO-1, ↑Tlr2, ↑Pla2g2, ↑Lyz1, ↑*Prevotella*	[94]
Other bioactivities	In vitro studies		
	LRA extracts	The MIC of *S. aureus* was 3.125 mg/mL, the number of *S. aureus* colonies was 4.88-log10 CFU/mL, the extracellular K^+^ concentration of *S. aureus* was 0.88 mmol/L, ↓intracellular protein of staphylococcus aureus	[95]
	RA extracts	The value of MIC of the *Bacillus* cereus, *Listeria monocytogenes, Escherichia coli, and Salmonella typhimurium* was 0.78, 3.12, 3.12, and 3.12mg/mL, respectively.	[96]
	RA extracts	*S. pneumoniae* (MBC 8.0 mg/mL) and *C. diphtheriae* (MBC 0.5 mg/mL), *M. catarrhalis* (MBC 0.015 mg/mL), *H. pylori* (MIC 8 mg/mL), *Neisseria meningitidis* (MIC 0.06 mg/mL).	[97]
	In vivo studies		
	LRA extracts on mouse fatigue model (100, 400, 900 mg/kg)	↑Glu, ↑SOD, ↓LDH, ↓MDA, ↓BUN	[98]
	LRA extracts on retinal damage induced by blue light exposure mice (50, 200 mg/kg)	↑CAT, ↑SOD, ↑GSH-Px, ↓ROS, ↓MDA, ↑Nrf2, ↑HO-1, ↑NQO1, ↓IL-6, ↓L-1β, ↓TNF-α, ↓VEGF-A, ↓p-IκBα/IκBα, ↓Caspase-3/Bax, ↑retina, ↑PSL, ↑INL, ↑ONL	[99]
	RA extracts on STZ-induced diabetes rats (35, 140 mg/kg for 6 weeks)	Improved the disorder and disarrangement in INL and ONL, ↓GRP78, ↓RPEC apoptosis, ↓PTP1B, ↓Caspase-1	[100]
	RA extracts on alcohol-induced hepatic injury mice (25, 50, 100 mg/kg)	↓ALT, ↓AST, ↓CHO, ↓LDL, ↓TBIL, ↓NF-κB, ↓TGF-β	[101]

↑ indicates the promotion trend, and ↓ indicates the downward trend.

## Data Availability

No new data were created or analyzed in this study. Data sharing is not applicable to this article.

## References

[1] Yao T., Thompson L.G., Mosbrugger V., Zhang F., Ma Y., Luo T., Xu B., Yang X., Joswiak D.R., Wang W. (2012). Third pole environment (TPE). Environ. Dev..

[2] Zhang B.-p., Chen X.-d., Li B.-l., Yao Y.-h. (2002). Biodiversity and conservation in the Tibetan Plateau. J. Geogr. Sci..

[3] Zhao Q., Zhang J., Li Y., Yang Z., Wang Q., Jia Q. (2024). Integrated Metabolomic and Transcriptomic Analysis of Nitraria Berries Indicate the Role of Flavonoids in Adaptation to High Altitude. Metabolites.

[4] Sun P., Hao R., Fan F., Wang Y., Zhu F. (2025). Adaptation of High-Altitude Plants to Plateau Abiotic Stresses: A Case Study of the Qinghai-Tibet Plateau. Int. J. Mol. Sci..

[5] Li X., Liu H., Li C., Li Y. (2024). A systematic review on the morphology structure, propagation characteristics, resistance physiology and exploitation and utilization of *Nitraria tangutorum* Bobrov. PeerJ.

[6] Liu Z., Dong B., Liu C., Zong Y., Shao Y., Liu B., Yue H. (2020). Variation of anthocyanin content in fruits of wild and cultivated *Lycium ruthenicum*. Ind. Crop. Prod..

[7] Liu C., Duan N., Chen X., Li X., Zhao N., Cao W., Li H., Liu B., Tan F., Zhao X. (2023). Transcriptome profiling and chlorophyll metabolic pathway analysis reveal the response of *Nitraria tangutorum* to increased nitrogen. Plants.

[8] Liu Z., Tang X., Liu C., Dong B., Shao Y., Liu B., Yue H. (2020). Ultrasonic extraction of anthocyanins from *Lycium ruthenicum* Murr. and its antioxidant activity. Food Sci. Nutr..

[9] Wang H., Li J., Tao W., Zhang X., Gao X., Yong J., Zhao J., Zhang L., Li Y., Duan J.-a. (2018). *Lycium ruthenicum* studies: Molecular biology, phytochemistry and pharmacology. Food Chem..

[10] Bueno J.M., Sáez-Plaza P., Ramos-Escudero F., Jiménez A.M., Fett R., Asuero A.G. (2012). Analysis and antioxidant capacity of anthocyanin pigments. Part II: Chemical structure, color, and intake of anthocyanins. Crit. Rev. Anal. Chem..

[11] Yeung A.W.K., Solka M., Jóźwik A., Ksepka N., Matin M., Wang D., Zielińska A., Mohanasundaram A., Vejux A., Zarrouk A. (2024). Anthocyanins-dietary natural products with a variety of bioactivities for the promotion of human and animal health. Anim. Sci. Pap. Rep..

[12] Gowd V., Jia Z.Q., Chen W. (2017). Anthocyanins as promising molecules and dietary bioactive components against diabetes A—Review of recent advances. Trends Food Sci. Technol..

[13] Wallace T.C., Giusti M.M. (2015). Anthocyanins. Adv. Nutr..

[14] Zeng S.H., Lin S., Wang Z.Q., Zong Y., Wang Y. (2024). The health-promoting anthocyanin petanin in fruit: A promising natural colorant. Crit. Rev. Food Sci. Nutr..

[15] Tena N., Martín J., Asuero A.G. (2020). State of the art of anthocyanins: Antioxidant activity, sources, bioavailability, and therapeutic effect in human health. Antioxidants.

[16] Chen W., Müller D., Richling E., Wink M. (2013). Anthocyanin-rich purple wheat prolongs the life span of *Caenorhabditis elegans* probably by activating the DAF-16/FOXO transcription factor. J. Agric. Food Chem..

[17] Les F., Cásedas G., Gómez C., Moliner C., Valero M.S., López V. (2021). The role of anthocyanins as antidiabetic agents: From molecular mechanisms to in vivo and human studies. J. Physiol. Biochem..

[18] Majhi S. (2022). Discovery, development and design of anthocyanins-inspired anticancer agents: A comprehensive review. Anti Cancer Agents Med. Chem. Anti Cancer Agents.

[19] Saini R.K., Khan M.I., Shang X., Kumar V., Kumari V., Kesarwani A., Ko E.-Y. (2024). Dietary sources, stabilization, health benefits, and industrial application of anthocyanins—A review. Foods.

[20] Tao L., Hao F., Fei P., Chen D., Fan H., Zhao S., Wang Y., Li B., Ma Y., Zhao X. (2022). Advance on Traditional Uses, Phytochemistry and Pharmacology of *Lycium ruthenicum* MURR. Pharm. Chem. J..

[21] Zheng J., Ding C., Wang L., Li G., Shi J., Li H., Wang H., Suo Y. (2011). Anthocyanins composition and antioxidant activity of wild *Lycium ruthenicum* Murr. from Qinghai-Tibet Plateau. Food Chem..

[22] Sang J., Ma Q., Li B., Li C.-Q. (2018). An approach for extraction, purification, characterization and quantitation of acylated-anthocyanins from *Nitraria tangutorun* Bobr. fruit. J. Food Meas. Charact..

[23] Zheng J., Li H., Ding C., Suo Y., Wang L., Wang H. (2011). Anthocyanins composition and antioxidant activity of two major wild *Nitraria tangutorun* Bobr. variations from Qinghai–Tibet Plateau. Food Res. Int..

[24] Chen F., Sun Y., Zhao G., Liao X., Hu X., Wu J., Wang Z. (2007). Optimization of ultrasound-assisted extraction of anthocyanins in red raspberries and identification of anthocyanins in extract using high-performance liquid chromatography–mass spectrometry. Ultrason. Sonochemistry.

[25] Schulz M., Chim J.F. (2019). Nutritional and bioactive value of *Rubus berries*. Food Biosci..

[26] You Q., Wang B., Chen F., Huang Z., Wang X., Luo P.G. (2011). Comparison of anthocyanins and phenolics in organically and conventionally grown blueberries in selected cultivars. Food Chem..

[27] Gonçalves A.C., Falcão A., Alves G., Lopes J.A., Silva L.R. (2022). Employ of anthocyanins in nanocarriers for nano delivery: In vitro and in vivo experimental approaches for chronic diseases. Pharmaceutics.

[28] da Silva F.L., Escribano-Bailón M.T., Alonso J.J.P., Rivas-Gonzalo J.C., Santos-Buelga C. (2007). Anthocyanin pigments in strawberry. LWT Food Sci. Technol..

[29] Šimerdová B., Bobríková M., Lhotská I., Kaplan J., Křenová A., Šatínský D. (2021). Evaluation of anthocyanin profiles in various blackcurrant cultivars over a three-year period using a fast HPLC-DAD method. Foods.

[30] Urbstaite R., Raudone L., Janulis V. (2022). Phytogenotypic anthocyanin profiles and antioxidant activity variation in fruit samples of the American cranberry (*Vaccinium macrocarpon Aiton*). Antioxidants.

[31] Martini S., Conte A., Tagliazucchi D. (2017). Phenolic compounds profile and antioxidant properties of six sweet cherry (Prunus avium) cultivars. Food Res. Int..

[32] Sharma R., Raghuvanshi R., Kumar R., Thakur M.S., Kumar S., Patel M.K., Chaurasia O., Saxena S. (2022). Current findings and future prospective of high-value trans Himalayan medicinal plant *Lycium ruthenicum* Murr: A systematic review. Clin. Phytoscience.

[33] Xu M.-L., He Y.-F., Xie L., Qu L.-B., Xu G.-R., Cui C.-X. (2024). Research progress on active ingredients and product development of *Lycium ruthenicum Murray*. Molecules.

[34] Liu Y., Deng Y., Yang Y., Dong H., Li L., Chen G. (2024). Comparison of different drying pretreatment combined with ultrasonic-assisted enzymolysis extraction of anthocyanins from *Lycium ruthenicum* Murr. Ultrason. Sonochemistry.

[35] Zhou X., Wu Y., Wang Y., Zhou X., Chen X., Xi J. (2022). An efficient approach for the extraction of anthocyanins from *Lycium ruthenicum* using semi-continuous liquid phase pulsed electrical discharge system. Innov. Food Sci. Emerg. Technol..

[36] Dong Y., Zhong W., Yang C., Zhang Y., Yang D. (2022). Study on anthocyanins from *Lycium ruthenicum* Murr via ultrasonic microwave synergistic extraction and its antioxidant properties. Front. Sustain. Food Syst..

[37] Shen M., Liu K., Liang Y., Liu G., Sang J., Li C. (2020). Extraction optimization and purification of anthocyanins from *Lycium ruthenicum* Murr. and evaluation of tyrosinase inhibitory activity of the anthocyanins. J. Food Sci..

[38] Sang J., Dang K.-k., Ma Q., Li B., Huang Y.-y., Li C.-q. (2018). Partition Behaviors of Different Polar Anthocyanins in Aqueous Two-Phase Systems and Extraction of Anthocyanins from *Nitraria tangutorun*Bobr. and *Lycium ruthenicum* Murr. Food Anal. Meth..

[39] Sang J., Ma Q., Ren M.-j., He S.-t., Feng D.-d., Yan X.-l., Li C.-q. (2018). Extraction and characterization of anthocyanins from *Nitraria tangutorun*bobr. dry fruit and evaluation of their stability in aqueous solution and taurine-contained beverage. J. Food Meas. Charact..

[40] Sang J., Liu K., Ma Q., Li B., Li C.-q. (2019). Combination of a deep eutectic solvent and macroporous resin for green recovery of anthocyanins from *Nitraria tangutorun*Bobr. fruit. Sep. Sci. Technol..

[41] Wang Y., Ye Y., Wang L., Yin W., Liang J. (2021). Antioxidant activity and subcritical water extraction of anthocyanin from raspberry process optimization by response surface methodology. Food Biosci..

[42] Xue H., Tan J., Li Q., Tang J., Cai X. (2020). Optimization ultrasound-assisted deep eutectic solvent extraction of anthocyanins from raspberry using response surface methodology coupled with genetic algorithm. Foods.

[43] Wen Y., Chen H., Zhou X., Deng Q., Zhao Y., Zhao C., Gong X. (2015). Optimization of the microwave-assisted extraction and antioxidant activities of anthocyanins from blackberry using a response surface methodology. Rsc Adv..

[44] Wu D., Gao T., Yang H., Du Y., Li C., Wei L., Zhou T., Lu J., Bi H. (2015). Simultaneous microwave/ultrasonic-assisted enzymatic extraction of antioxidant ingredients from *Nitraria tangutorun*Bobr. juice by-products. Ind. Crop. Prod..

[45] Abu Bakar M.F., Ismail N.A., Isha A., Mei Ling A.L. (2016). Phytochemical composition and biological activities of selected wild berries (*Rubus moluccanus* L., *R. fraxinifolius Poir*., and *R. alpestris Blume*). Evid. Based Complement. Altern. Med..

[46] Yan Y., Nisar T., Fang Z., Wang L., Wang Z., Gu H., Wang H., Wang W. (2022). Current developments on chemical compositions, biosynthesis, color properties and health benefits of black goji anthocyanins: An updated review. Horticulturae.

[47] He J., Giusti M.M. (2010). Anthocyanins: Natural colorants with health-promoting properties. Annu. Rev. Food Sci. Technol..

[48] Jin H., Liu Y., Yang F., Wang J., Fu D., Zhang X., Peng X., Liang X. (2015). Characterization of anthocyanins in wild *Lycium ruthenicum Murray* by HPLC-DAD/QTOF-MS/MS. Anal. Methods.

[49] Tian Z., Aierken A., Pang H., Du S., Feng M., Ma K., Gao S., Bai G., Ma C. (2016). Constituent analysis and quality control of anthocyanin constituents of dried *Lycium ruthenicum Murray* fruits by HPLC–MS and HPLC–DAD. J. Liq. Chromatogr. Relat. Technol..

[50] Chen S., Zhou H., Zhang G., Meng J., Deng K., Zhou W., Wang H., Wang Z., Hu N., Suo Y. (2019). Anthocyanins from *Lycium ruthenicum* Murr. ameliorated d-galactose-induced memory impairment, oxidative stress, and neuroinflammation in adult rats. J. Agric. Food Chem..

[51] Sang J., Zhang Y., Sang J., Li C.Q. (2019). Anthocyanins from Nitraria tangutorun: Qualitative and quantitative analyses, antioxidant and anti-inflammatory activities and their stabilities as affected by some phenolic acids. J. Food Meas. Charact..

[52] Zhang M., Ma J., Bi H., Song J., Yang H., Xia Z., Du Y., Gao T., Wei L. (2017). Characterization and cardioprotective activity of anthocyanins from *Nitraria tangutorum* Bobr. by-products. Food Funct..

[53] Chen S., Zhou H., Zhang G., Dong Q., Wang Z., Wang H., Hu N. (2021). Characterization, antioxidant, and neuroprotective effects of anthocyanins from *Nitraria tangutorum* Bobr. fruit. Food Chem..

[54] Hu N., Zheng J., Li W., Suo Y. (2014). Isolation, stability, and antioxidant activity of anthocyanins from *Lycium ruthenicum Murray* and *Nitraria tangutorum* Bobr of Qinghai-Tibetan plateau. Sep. Sci. Technol..

[55] de Ancos B., Gonzalez E., Cano M.P. (1999). Differentiation of raspberry varieties according to anthocyanin composition. Z. Für Leb. Und Forsch. A.

[56] Chen S., Jia Y., Wu Y., Ren F. (2024). Anthocyanin and its Bioavailability, Health Benefits, and Applications: A Comprehensive Review. Food Rev. Int..

[57] Teng H., Fang T., Lin Q., Song H., Liu B., Chen L. (2017). Red raspberry and its anthocyanins: Bioactivity beyond antioxidant capacity. Trends Food Sci. Tech..

[58] Tian Q., Giusti M.M., Stoner G.D., Schwartz S.J. (2006). Characterization of a new anthocyanin in black raspberries (*Rubus occidentalis*) by liquid chromatography electrospray ionization tandem mass spectrometry. Food Chem..

[59] Čanadanović-Brunet J., Vulić J., Ćebović T., Ćetković G., Čanadanović V., Djilas S., Šaponjac V.T. (2017). Phenolic profile, antiradical and antitumour evaluation of raspberries pomace extract from Serbia. Iran. J. Pharm. Res..

[60] Tanaka Y., Sasaki N., Ohmiya A. (2008). Biosynthesis of plant pigments: Anthocyanins, betalains and carotenoids. Plant J..

[61] Nistor M., Pop R., Daescu A., Pintea A., Socaciu C., Rugina D. (2022). Anthocyanins as key phytochemicals acting for the prevention of metabolic diseases: An overview. Molecules.

[62] Nguyen H.M., Putterill J., Dare A.P., Plunkett B.J., Cooney J., Peng Y., Souleyre E.J., Albert N.W., Espley R.V., Günther C.S. (2023). Two genes, ANS and UFGT2, from *Vaccinium* spp. are key steps for modulating anthocyanin production. Front. Plant Sci..

[63] Cai T., Ge-Zhang S., Song M. (2023). Anthocyanins in metabolites of purple corn. Front. Plant Sci..

[64] Yonekura-Sakakibara K., Nakayama T., Yamazaki M., Saito K. (2009). Modification and stabilization of anthocyanins. Anthocyanins: Biosynthesis, Functions, and Applications.

[65] Hichri I., Barrieu F., Bogs J., Kappel C., Delrot S., Lauvergeat V. (2011). Recent advances in the transcriptional regulation of the flavonoid biosynthetic pathway. J. Exp. Bot..

[66] Fuglevand G., Jackson J.A., Jenkins G.I. (1996). UV-B, UV-A, and blue light signal transduction pathways interact synergistically to regulate chalcone synthase gene expression in Arabidopsis. Plant Cell.

[67] Li P., Li Y.J., Zhang F.J., Zhang G.Z., Jiang X.Y., Yu H.M., Hou B.K. (2017). The Arabidopsis UDP-glycosyltransferases UGT79B2 and UGT79B3, contribute to cold, salt and drought stress tolerance via modulating anthocyanin accumulation. Plant J..

[68] Gou J.-Y., Felippes F.F., Liu C.-J., Weigel D., Wang J.-W. (2011). Negative regulation of anthocyanin biosynthesis in *Arabidopsis* by a miR156-targeted SPL transcription factor. Plant Cell.

[69] Chen S., Hu N., Wang H., Wu Y., Li G. (2022). Bioactivity-guided isolation of the major anthocyanin from *Lycium ruthenicum* Murr. fruit and its antioxidant activity and neuroprotective effects in vitro and in vivo. Food Funct..

[70] Tang J., Yan Y., Ran L., Mi J., Sun Y., Lu L., Gao Y., Zeng X., Cao Y. (2017). Isolation, antioxidant property and protective effect on PC12 cell of the main anthocyanin in fruit of *Lycium ruthenicum Murray*. J. Funct. Foods.

[71] Ma T., Hu N., Ding C., Zhang Q., Li W., Suo Y., Wang H., Bai B., Ding C. (2016). In vitro and in vivo biological activities of anthocyanins from *Nitraria tangutorun*Bobr. fruits. Food Chem..

[72] Peng Y., Yan Y., Wan P., Dong W., Huang K., Ran L., Mi J., Lu L., Zeng X., Cao Y. (2020). Effects of long-term intake of anthocyanins from *Lycium ruthenicum Murray* on the organism health and gut microbiota in vivo. Food Res. Int..

[73] Fan H., Ji Y., Wang Y., Liu D., Wei T., Cao X., Yang M., Bai C., Wang Z. (2022). Anthocyanins from *Lycium ruthenicum* murray inhibit HepG2 cells growth, metastasis and promote apoptosis and G2/M phase cycle arrest by activating the AMPK/mTOR autophagy pathway. Evid. Based Complement. Altern. Med..

[74] Qin X.S., Wang X.Y., Xu K., Yang X.B., Wang Q., Liu C., Wang X.K., Guo X., Sun J.Y., Li L. (2022). Synergistic antitumor effects of polysaccharides and anthocyanins from *Lycium ruthenicum* Murr. on human colorectal carcinoma LoVo cells and the molecular mechanism. Food Sci. Nutr..

[75] Chen L., Li M., Zhou H., Liu Y., Pang W., Ma T., Niu C., Yang Z., Chang A.K., Li X. (2023). Sirtuin1 (SIRT1) is involved in the anticancer effect of black raspberry anthocyanins in colorectal cancer. Eur. J. Nutr..

[76] Ding M., Feng R., Wang S.Y., Bowman L., Lu Y., Qian Y., Castranova V., Jiang B.-H., Shi X. (2006). Cyanidin-3-glucoside, a natural product derived from blackberry, exhibits chemopreventive and chemotherapeutic activity. J. Biol. Chem..

[77] Wang L.W., Wan G.M., Wang G., Zhang M.H., Li N.X., Zhang Q.N., Yan H.L. (2022). Anthocyanin from *Lycium ruthenicum* Murr. in the Qaidam Basin Alleviates Ultraviolet-Induced Apoptosis of Human Skin Fibroblasts by Regulating the Death Receptor Pathway. Clin. Cosmet. Investig. Dermatol..

[78] Gao W., Wang Y.-s., Hwang E., Lin P., Bae J., Seo S.A., Yan Z., Yi T.-H. (2018). *Rubus idaeus* L. (red raspberry) blocks UVB-induced MMP production and promotes type I procollagen synthesis via inhibition of MAPK/AP-1, NF-κβ and stimulation of TGF-β/Smad, Nrf2 in normal human dermal fibroblasts. J. Photochem. Photobiol. B Biol..

[79] Chen S., Wang H., Hu N. (2022). Long-term dietary *Lycium ruthenicum* murr. Anthocyanins intake alleviated oxidative stress-mediated aging-related liver injury and abnormal amino acid metabolism. Foods.

[80] Wang R., Ren L., Wang Y., Hu N., Tie F., Dong Q., Wang H. (2024). Multi-Protective Effects of Petunidin-3-O-(trans-p-coumaroylrutinoside)-5-O-glucoside on D-Gal-Induced Aging Mice. Int. J. Mol. Sci..

[81] Wang Z., Sun L., Fang Z., Nisar T., Zou L., Li D., Guo Y. (2021). *Lycium ruthenicum Murray* anthocyanins effectively inhibit α-glucosidase activity and alleviate insulin resistance. Food Biosci..

[82] Ren L., Dong Q., Liu Z., Wang Y., Tan N., Wang H., Hu N. (2024). Optimization of subcritical water extraction, UPLC–triple–TOF–MS/MS analysis, antioxidant and α-glucosidase inhibitory activities of anthocyanins from *Nitraria sibirica* Pall. fruits. Food Chem. X.

[83] Tian B., Zhao J., Xie X., Chen T., Yin Y., Zhai R., Wang X., An W., Li J. (2021). Anthocyanins from the fruits of *Lycium ruthenicum Murray* improve high-fat diet-induced insulin resistance by ameliorating inflammation and oxidative stress in mice. Food Funct..

[84] Ren L., Tan N., Ouyang J., Wang R., Tie F., Dong Q., Wang H., Hu N. (2024). Hypoglycaemic activity of the anthocyanin enriched fraction of *Lycium ruthenicum* Murr. Fruits and its ingredient identification via UPLC–triple-TOF-MS/MS. Food Chem..

[85] Pergola C., Rossi A., Dugo P., Cuzzocrea S., Sautebin L. (2006). Inhibition of nitric oxide biosynthesis by anthocyanin fraction of blackberry extract. Nitric Oxide.

[86] Szymanowska U., Baraniak B., Bogucka-Kocka A. (2018). Antioxidant, anti-inflammatory, and postulated cytotoxic activity of phenolic and anthocyanin-rich fractions from polana raspberry (*Rubus idaeus* L.) fruit and juice—In vitro study. Molecules.

[87] Zhang G., Chen S., Zhou W., Meng J., Deng K., Zhou H., Hu N., Suo Y. (2019). Anthocyanin composition of fruit extracts from *Lycium ruthenicum* and their protective effect for gouty arthritis. Ind. Crops Prod..

[88] Peng Y.J., Yan Y.M., Wan P., Chen D., Ding Y., Ran L.W., Mi J., Lu L., Zhang Z.J., Li X.Y. (2019). Gut microbiota modulation and anti-inflammatory properties of anthocyanins from the fruits of Murray in dextran sodium sulfate-induced colitis in mice. Free Radic. Biol. Med..

[89] Mu T., Guan Y., Chen T., Wang S., Li M., Chang A.K., Yang Z., Bi X. (2021). Black raspberry anthocyanins protect BV2 microglia from LPS-induced inflammation through down-regulating NOX2/TXNIP/NLRP3 signaling. J. Berry Res..

[90] Ma H., Johnson S.L., Liu W., DaSilva N.A., Meschwitz S., Dain J.A., Seeram N.P. (2018). Evaluation of polyphenol anthocyanin-enriched extracts of blackberry, black raspberry, blueberry, cranberry, red raspberry, and strawberry for free radical scavenging, reactive carbonyl species trapping, anti-glycation, anti-β-amyloid aggregation, and microglial neuroprotective effects. Int. J. Mol. Sci..

[91] Yan Y., Peng Y., Tang J., Mi J., Lu L., Li X., Ran L., Zeng X., Cao Y. (2018). Effects of anthocyanins from the fruit of *Lycium ruthenicum Murray* on intestinal microbiota. J. Funct. Foods.

[92] Peng Y., Yan Y., Wan P., Chen C., Chen D., Zeng X., Cao Y. (2021). Prebiotic effects in vitro of anthocyanins from the fruits of *Lycium ruthenicum Murray* on gut microbiota compositions of feces from healthy human and patients with inflammatory bowel disease. Lwt.

[93] Liu P., Zhou W., Xu W., Peng Y., Yan Y., Lu L., Mi J., Zeng X., Cao Y. (2021). The main anthocyanin monomer from *Lycium ruthenicum Murray* fruit mediates obesity via modulating the gut microbiota and improving the intestinal barrier. Foods.

[94] Su H., Xie L., Xu Y., Ke H., Bao T., Li Y., Chen W. (2019). Pelargonidin-3-O-glucoside derived from wild raspberry exerts antihyperglycemic effect by inducing autophagy and modulating gut microbiota. J. Agric. Food Chem..

[95] Dong Y., Yang C., Zhong W., Shu Y., Zhang Y., Yang D. (2022). Antibacterial effect and mechanism of anthocyanin from *Lycium ruthenicum* Murr. Front. Microbiol..

[96] Vara A.L., Pinela J., Dias M.I., Petrović J., Nogueira A., Soković M., Ferreira I.C., Barros L. (2020). Compositional features of the “Kweli” red raspberry and its antioxidant and antimicrobial activities. Foods.

[97] Krauze-Baranowska M., Majdan M., Hałasa R., Głód D., Kula M., Fecka I., Orzeł A. (2014). The antimicrobial activity of fruits from some cultivar varieties of *Rubus idaeus* and *Rubus occidentalis*. Food Funct..

[98] Zhang M.J., Xing L.J., Wang Y., Luo R.F., Li X.Y., Dong J. (2022). Anti-fatigue activities of anthocyanins from *Lycium ruthenicum* Murry. Food Sci. Technol..

[99] Li P., Li Z., Sun Q., Zhang W., Huang X., Si M., Du X., Wang S. (2024). Protective effect and mechanism of *Lycium ruthenicum Murray* anthocyanins against retinal damage induced by blue light exposure. J. Food Sci..

[100] Xiao T., Zhi Y., Tian F., Huang F., Cheng X., Wu A., Tao L., Guo Z., Shen X. (2023). Ameliorative effect of black raspberry anthocyanins on diabetes retinopathy by inhibiting axis protein tyrosine phosphatase 1B-endoplasmic reticulum stress. J. Funct. Foods.

[101] Xiao T., Luo Z., Guo Z., Wang X., Ding M., Wang W., Shen X., Zhao Y. (2021). Multiple roles of black raspberry anthocyanins protecting against alcoholic liver disease. Molecules.

[102] Ighodaro O., Akinloye O. (2018). First line defence antioxidants-superoxide dismutase (SOD), catalase (CAT) and glutathione peroxidase (GPX): Their fundamental role in the entire antioxidant defence grid. Alex. J. Med..

[103] Reuter S., Gupta S.C., Chaturvedi M.M., Aggarwal B.B. (2010). Oxidative stress, inflammation, and cancer: How are they linked?. Free Radic. Biol. Med..

[104] Chen W., Su H., Xu Y., Bao T., Zheng X. (2016). Protective effect of wild raspberry (*Rubus hirsutus* Thunb.) extract against acrylamide-induced oxidative damage is potentiated after simulated gastrointestinal digestion. Food Chem..

[105] Demain A.L., Vaishnav P. (2011). Natural products for cancer chemotherapy. Microb. Biotechnol..

[106] Gavas S., Quazi S., Karpiński T.M. (2021). Nanoparticles for cancer therapy: Current progress and challenges. Nanoscale Res. Lett..

[107] Zhang L., Lou W., Wang J. (2024). Advances in nucleic acid therapeutics: Structures, delivery systems, and future perspectives in cancer treatment. Clin. Exp. Med..

[108] Wang L.-S., Stoner G.D. (2008). Anthocyanins and their role in cancer prevention. Cancer Lett..

[109] Lin B.W., Gong C.C., Song H.F., Cui Y.Y. (2017). Effects of anthocyanins on the prevention and treatment of cancer. Br. J. Pharmacol..

[110] Rashwan A.K., Karim N., Xu Y., Xie J., Cui H., Mozafari M., Chen W. (2023). Potential micro-/nano-encapsulation systems for improving stability and bioavailability of anthocyanins: An updated review. Crit. Rev. Food Sci. Nutr..

[111] Flores G., del Castillo M.L.R., Costabile A., Klee A., Guergoletto K.B., Gibson G.R. (2015). In vitro fermentation of anthocyanins encapsulated with cyclodextrins: Release, metabolism and influence on gut microbiota growth. J. Funct. Foods.

[112] Chung C., Rojanasasithara T., Mutilangi W., McClements D.J. (2015). Enhanced stability of anthocyanin-based color in model beverage systems through whey protein isolate complexation. Food Res. Int..

[113] Zhang L., Wang Y., Cao Y., Wang F., Li F. (2025). Enhancing the Bioavailability and Stability of Anthocyanins for the Prevention and Treatment of Central Nervous System-Related Diseases. Foods.

[114] Wang B., Tang X., Mao B., Zhang Q., Tian F., Zhao J., Cui S., Chen W. (2024). Anti-aging effects and mechanisms of anthocyanins and their intestinal microflora metabolites. Crit. Rev. Food Sci..

[115] Chen P.-D., Li J.-J., Zhang S., Chen D.-X., Chen X., Yin Z.-C., Shen Y.-P., Gao J.-Y., Zhang J.-K., Chen H.-B. (2025). Research progress on the regulatory effects of Chinese food and medicine homology on type 1 diabetes mellitus. Food Med. Homol..

[116] Sancho R.A.S., Pastore G.M. (2012). Evaluation of the effects of anthocyanins in type 2 diabetes. Food Res. Int..

[117] Sharma A., Lee H.-J. (2022). Anti-inflammatory activity of bilberry (*Vaccinium myrtillus* L.). Curr. Issues Mol. Biol..

[118] Ma Z., Du B., Li J., Yang Y., Zhu F. (2021). An insight into anti-inflammatory activities and inflammation related diseases of anthocyanins: A review of both in vivo and in vitro investigations. Int. J. Mol. Sci..

[119] Szymanowska U., Baraniak B. (2019). Antioxidant and potentially anti-inflammatory activity of anthocyanin fractions from pomace obtained from enzymatically treated raspberries. Antioxidants.

[120] Li P., Feng D., Yang D., Li X., Sun J., Wang G., Tian L., Jiang X., Bai W. (2021). Protective effects of anthocyanins on neurodegenerative diseases. Trends Food Sci. Technol..

[121] Zhong H., Xu J., Yang M., Hussain M., Liu X., Feng F., Guan R. (2023). Protective effect of anthocyanins against neurodegenerative diseases through the microbial-intestinal-brain axis: A critical review. Nutrients.

[122] Badshah H., Kim T.H., Kim M.O. (2015). Protective effects of anthocyanins against amyloid beta-induced neurotoxicity in vivo and in vitro. Neurochem. Int..

[123] Faria A., Fernandes I., Norberto S., Mateus N., Calhau C. (2014). Interplay between anthocyanins and gut microbiota. J. Agric. Food Chem..

[124] Tian L., Tan Y., Chen G., Wang G., Sun J., Ou S., Chen W., Bai W. (2019). Metabolism of anthocyanins and consequent effects on the gut microbiota. Crit. Rev. Food Sci. Nutr..

[125] Gamage G.C.V., Goh J.K., Choo W.S. (2024). Application of anthocyanins from black goji berry in fermented dairy model food systems: An alternate natural purple color. LWT.

[126] Rocha R., Pinela J., Abreu R.M., Añibarro-Ortega M., Pires T.C., Saldanha A.L., Alves M.J., Nogueira A., Ferreira I.C., Barros L. (2020). Extraction of anthocyanins from red raspberry for natural food colorants development: Processes optimization and in vitro bioactivity. Processes.

[127] Patras A., Brunton N.P., O’Donnell C., Tiwari B.K. (2010). Effect of thermal processing on anthocyanin stability in foods; mechanisms and kinetics of degradation. Trends Food Sci. Technol..

[128] Yousuf B., Gul K., Wani A.A., Singh P. (2016). Health benefits of anthocyanins and their encapsulation for potential use in food systems: A review. Crit. Rev. Food Sci. Nutr..

[129] Sudharsan M.S., Mani H., Kumar L., Pazhamalai V., Hari S. (2023). Pectin based colorimetric film for monitoring food freshness. Curr. Res. Nutr. Food Sci. J..

[130] Qin Y., Yun D., Xu F., Chen D., Kan J., Liu J. (2021). Smart packaging films based on starch/polyvinyl alcohol and *Lycium ruthenicum* anthocyanins-loaded nano-complexes: Functionality, stability and application. Food Hydrocoll..

[131] Liu B., Gao J., Liu X., Zhang X., Zeng X., Zhang X., Zhao P. (2024). Preparation of soybean isolate protein/xanthan gum/agar-*Lycium ruthenicum* anthocyanins intelligent indicator films and its application in mutton preservation. Int. J. Biol. Macromol..

[132] Zhao Y., Gao L., Wang J., Xue Z., Zhang M., Ma X., Wang G., Lv S. (2023). Preparation and Application of pH-Sensitive Film Containing Anthocyanins Extracted from *Lycium ruthenicum* Murr. Materials.

[133] Albert C., Codină G.G., Héjja M., András C.D., Chetrariu A., Dabija A. (2022). Study of antioxidant activity of garden blackberries (*Rubus fruticosus* L.) extracts obtained with different extraction solvents. Appl. Sci..

[134] Zhou L., Liu Z., Wang W., Yao M., Wang J., Liu Y., Liu Z. (2025). Anthocyanins in Black Wolfberry (*Lycium ruthenicum Murray*): Chemical Structure, Composition, Stability and Biological Activity. Food Rev. Int..

[135] Kozłowska A., Dzierżanowski T. (2021). Targeting inflammation by anthocyanins as the novel therapeutic potential for chronic diseases: An update. Molecules.

[136] Bars-Cortina D., Sakhawat A., Piñol-Felis C., Motilva M.-J. (2022). Chemopreventive effects of anthocyanins on colorectal and breast cancer: A review. Seminars in Cancer Biology.

[137] Shrestha B., Pandey R.P., Darsandhari S., Parajuli P., Sohng J.K. (2019). Combinatorial approach for improved cyanidin 3-O-glucoside production in Escherichia coli. Microb. Cell Factories.

[138] Cheng Y., Liu J., Li L., Ren J., Lu J., Luo F. (2023). Advances in embedding techniques of anthocyanins: Improving stability, bioactivity and bioavailability. Food Chem. X.

[139] Chen B.-H., Stephen Inbaraj B. (2019). Nanoemulsion and nanoliposome based strategies for improving anthocyanin stability and bioavailability. Nutrients.

[140] Xue H., Sang Y., Gao Y., Zeng Y., Liao J., Tan J. (2022). Research progress on absorption, metabolism, and biological activities of anthocyanins in berries: A review. Antioxidants.

